# Seasonal pattern of food habits of large herbivores in riverine alluvial grasslands of Brahmaputra floodplains, Assam

**DOI:** 10.1038/s41598-021-04295-4

**Published:** 2022-01-10

**Authors:** Anita Devi, Syed Ainul Hussain, Monika Sharma, Govindan Veeraswami Gopi, Ruchi Badola

**Affiliations:** Wildlife Institute of India, Chandrabani, Post Box # 18, Dehra Dun, Uttarakhand 248001 India

**Keywords:** Behavioural ecology, Community ecology

## Abstract

Jarman–Bell (1974) hypothesized that in the dry savanna of Africa, small-bodied herbivores tend to browse more on forage with high protein and low fibre content. This implies browsing on high nutritive forage by meso-herbivores, and grazing and mixed feeding on coarse forage by mega-herbivores. We tested this hypothesis in the riverine alluvial grasslands of the Kaziranga National Park (KNP), where seasonal flood and fire play an important role in shaping the vegetation structure. We analyzed the feeding habits and quality of major forage species consumed by three mega-herbivores, viz*.* greater one-horned rhino, Asian elephant, and Asiatic wild buffalo, and three meso-herbivores, viz*.* swamp deer, hog deer, and sambar. We found that both mega and meso-herbivores were grazers and mixed feeders. Overall, 25 forage plants constituted more than 70% of their diet. Among monocots, family Poaceae with *Saccharum* spp*.* (contributing > 9% of the diet), and, among dicots, family Rhamnaceae with *Ziziphus jujuba* (contributing > 4% of the diet) fulfilled the dietary needs. In the dry season, the concentration of crude protein, neutral detergent fibre, calcium, sodium, and phosphorous varied significantly between monocots and dicots, whereas only calcium and sodium concentrations varied significantly in the wet season. Dicots were found to be more nutritious throughout the year. Compared to the dry season, the monocots, viz. *Alpinia* *nigra*, *Carex vesicaria*, *Cynodon dactylon*, *Echinochloa crus-galli*, *Hemarthria compressa*, *Imperata cylindrica*, and *Saccharum* spp*.*, with their significantly high crude protein, were more nutritious during the wet season. Possibly due to the availability of higher quality monocots in the wet season, both mega and meso-herbivores consume it in high proportion. We concluded that the Jarman–Bell principle does not apply to riverine alluvial grasslands as body size did not explain the interspecific dietary patterns of the mega and meso-herbivores. This can be attributed to seasonal floods, habitat and forage availability, predation risk, and management practices such as controlled burning of the grasslands. The ongoing succession and invasion processes, anthropogenic pressures, and lack of grassland conservation policy are expected to affect the availability of the principal forage and suitable habitat of large herbivores in the Brahmaputra floodplains, which necessitates wet grassland-based management interventions for the continued co-existence of large herbivores in such habitats.

## Introduction

Understanding the niche of an animal is crucial for understanding the community dynamics of dependent consumers. Foraging is one of the fundamental elements of a niche^1,2^. In sympatric herbivores, the foraging patterns provide insights into the utilization patterns of the occupied habitat, which is important for making assumptions about the behaviour, physiology, morphology, and population dynamics of the predators, prey, and competitors^3^. In the early 1960s, the ecological succession and separation theory described forage resource partitioning as the reason for the co-existence of herbivores of different body sizes^4–6^. Bell^7^ and Jarman^8^ explained the co-existence of mega and meso-herbivores as a function of body mass and digestive physiology, whereas Hofmann and Stewart^9^ reasoned digestive physiology as the explanation for foraging style. The Jarman–Bell principle emphasized that the quality and quantity of large herbivore diet correlates with their body size; specifically, the diet quality decreases as body size increases. Depending on body size, herbivores consume a large or small amount of coarse forage to fulfil their body requirement^8,10^. The allometric theory^11^ and digestive physiology^12^ explained the mechanism behind the Jarman–Bell principle. The allometric theory explained that the length of the digestive tract is directly proportional to the body mass, and consequently, the metabolism rate is inversely proportional to the body mass. Demment and Van Soest^12^ provided evidence that the capability to digest coarse forage increases with the increase in gut capacity. Hence, the digestive capability of mega and meso-herbivores plays a crucial role in their dietary selection^13,14^. Thus, depending on both nutritional (crude protein content, mineral content, and digestibility) and anti-nutritional parameters (plant secondary metabolites or fibre), meso-herbivores need to browse on forage with high protein and low fibre content. In contrast, mega-herbivores may feed on forage with low protein and high fibre content^15,16^.

The preponderance of the studies that examined the ecology of large assemblages of sympatric herbivores and tested the body mass principle have primarily emerged from Africa and North America^17,18^. The studies conducted to test the Jarman–Bell principle in the African savannas^19–24^ and the protected areas of North America^21^ primarily covered dry tropical grasslands, forests, or savannas. These studies provided insight into resource segregation, competition, and habitat utilization along the temporal^19,20^, and spatial gradients^21–25^.

Of the 19 terrestrial mammalian herbivore species with a body mass greater than 100 kg, in South and South-East Asia, 14 are found in India^26^. Though the herbivore species found in India are distinct from those in Africa, the similarities in the diverse range of body sizes, from mega-herbivores like elephant, with a body weight of 3000 to 5400 kg, to meso-herbivores like mouse deer, with a body weight of 2 to 4 kg, provide the opportunity to test the Jarman–Bell principle^6,27,28^. The literature available to understand the science of wild large herbivore foraging ecology at the community level is limited from Asia^29^. Most of the research conducted in India studied up to four wild herbivore species and contributed mostly to their biology and ecology^6,29^. Ahrestani^6^ tested the Jarman–Bell principle for chital (*Axis axis*), sambar (*Rusa unicolor*), gaur (*Bos gaurus*), and elephant (*Elephas maximus*) in the dry tropical forests of India, where he concluded that body size does not explain the graze to browse ratio of sambar and chital. Wegge et al.^29^ tested the Jarman–Bell principle for rhinoceros (*Rhinoceros unicornis*), swamp deer (*Rucervus duvaucelii*), and hog deer (*Axis porcinus*) in riverine alluvial grasslands of Nepal, where they concluded that the body size does not explain the consumption of higher graminoids by smaller herbivores. There is little understanding of how this principle explains the foraging pattern and resource partitioning among large herbivores along the temporal and spatial gradients in the riverine alluvial grassland ecosystem. Large assemblages of mega and meso-herbivores in the Brahmaputra valley provide an opportunity to examine the applicability of the Jarman–Bell principle in a moist grassland ecosystem, which is subjected to anthropogenic pressure and is vulnerable to climate change.

In the last few decades, climate change and habitat loss have impacted biological systems. It is estimated that since 1970, 58% of animal populations have faced the threat of extinction^30–32^. Climate change poses a serious threat to herbivores directly by influencing rainfall and temperature, and indirectly through the occurrence of extreme climatic events such as fires, floods, and droughts, which may affect the availability and quality of forage and threaten their fitness, survival, migration, and reproductive success^33,34^. Globally, riverine alluvial grasslands in floodplains are threatened, primarily due to fragmentation and degradation of such habitats^35^. In the Brahmaputra valley, remnant riverine alluvial grasslands found mainly in and around protected areas are restricted in their spatial extent thereby, limiting the range of obligate large herbivores^35,36^. The decreasing trend of riverine alluvial grasslands has resulted in low species richness of large herbivore assemblages; consequently, the sample size to validate community ecology theory is often inadequate, which prompts this study^35^.

Based on feeding styles, herbivores are generally categorized as grazers (feeding mainly on graminoids or monocots), browsers (feeding mainly on browse or dicots), or mixed feeders (feeding on both monocots and dicots, according to their availability). Any change in the consumption of monocots (grazing) and dicots (browsing) results in changed diet compositions. Experimental studies conducted in the Serengeti-Mara ecosystem in the last 100 years have highlighted the importance of mega and meso-herbivores in converting open grassland to dense woodland and back to grassland^37^. Thus, in succession, both the absence and presence of mega and meso-herbivores plays an important role^38,39^. Browsers and mixed-feeders generally affect the savannas of Africa^40^.

**Figure 1:**
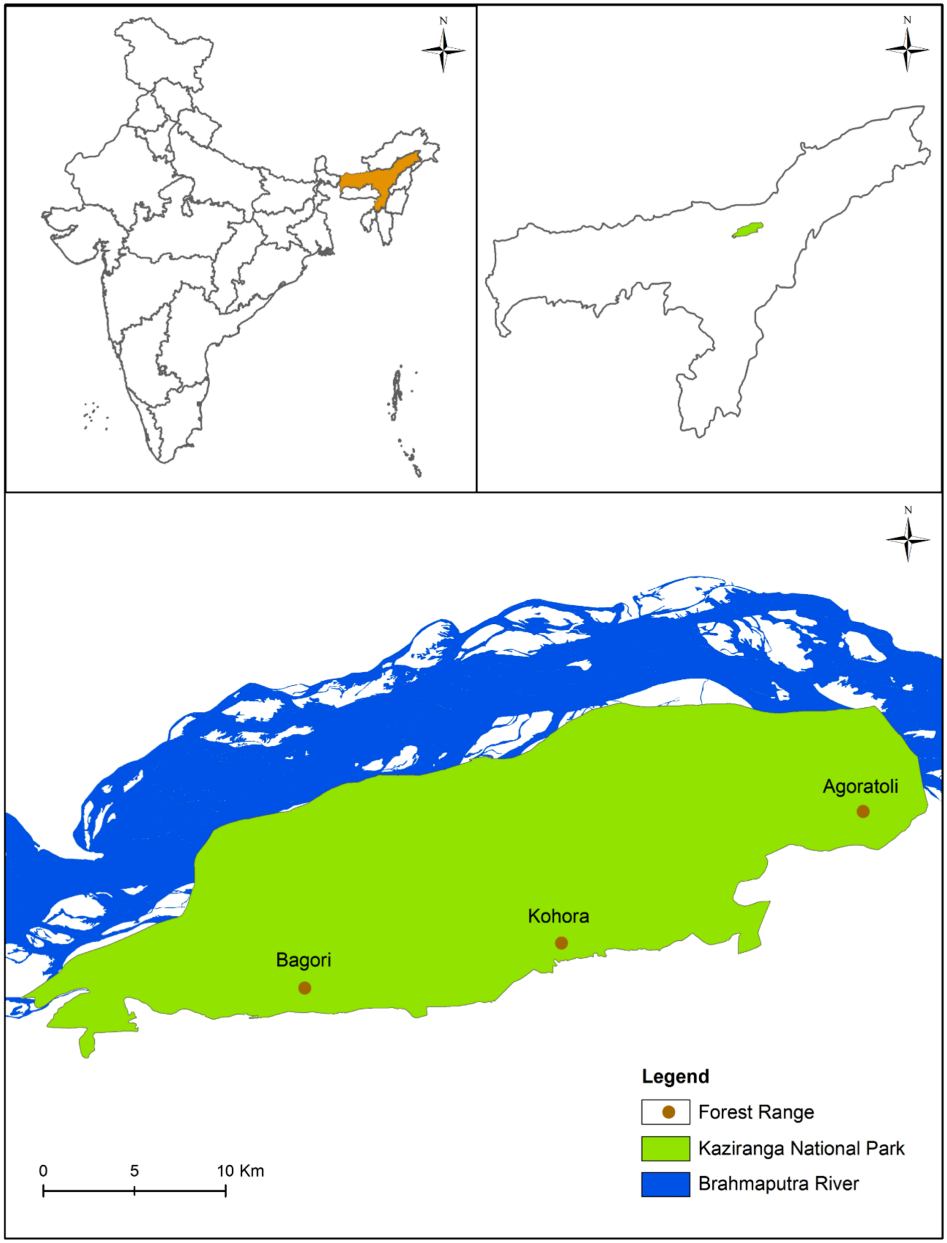
Map showing the location of the riverine alluvial grasslands in Kaziranga National Park, Assam. The map was created using ArcGIS v.10.2.2 software developed by ESRI (https://www.esri.com).

Even though the Brahmaputra floodplains in India harbour a large assemblage of mega and meso-herbivores, there is a dearth of studies on their community ecology. The limited information that is available is from Pobitora Wildlife Sanctuary^41,42^, Rajiv Gandhi Orang National Park^43^, Manas National Park^44^, and Kaziranga National Park (KNP)^45–48^, and is based on two to three species. Besides, there is little understanding of the habitat dynamics and the impact of seasonal change on the moist alluvial grasslands and their associated fauna. The riverine alluvial grasslands of KNP in the Brahmaputra floodplains are one of the few strongholds of several threatened species including the greater one-horned rhino (*R. unicornis*), Asian elephant (*E. maximus*), Asiatic wild buffalo (*Bubalus arnee*), swamp deer (*R. duvaucelii*), hog deer (*A. porcinus*), and sambar (*R. unicolor*), which necessitates their conservation (Fig. 1). This study was carried out to gain insight into how the body mass principle explains the co-existence of these six mega and meso-herbivores in riverine alluvial grasslands with respect to their diet composition and nutritional quality of principal forage. For the present study, based on the literature on body size and diet composition, the less selective mega-herbivores with very large body sizes (> 1000 kg), viz. rhino, elephant, and buffalo, were categorized as coarse feeders^13,49^. Whereas the more selective meso-herbivores with small to medium body sizes (> 5 kg and < 500 kg), viz. swamp deer, hog deer, and sambar, were categorized as soft feeders^14,50,51^.

Based on the information from riverine alluvial grasslands^18,29^, we predicted that both mega and meso-herbivores would consume more graze-based diet in the wet season (H_1_), and compared to dry season, the principal monocot forage of mega and meso-herbivores would be more nutritious in the wet season (H_2_). This study aims to provide an overview of the feeding habits of mega and meso-herbivores with the following research questions: (1) is there any difference in the diet compositions of the mega and meso-herbivores in each season in terms of (a) monocot and dicot, (b) the six categories of growth form, viz. grass, sedge, herb, shrub, climber and tree, and (c) the forage plant species contributing to the diets of the mega and meso-herbivores; (2) is there any difference in the seasonal nutrient parameters of major forage plants consumed by mega and meso-herbivores; and (3) which nutrient factors govern the forage utilization by mega and meso-herbivores.

## Results

### Dietary spectrum

**Figure 2:**
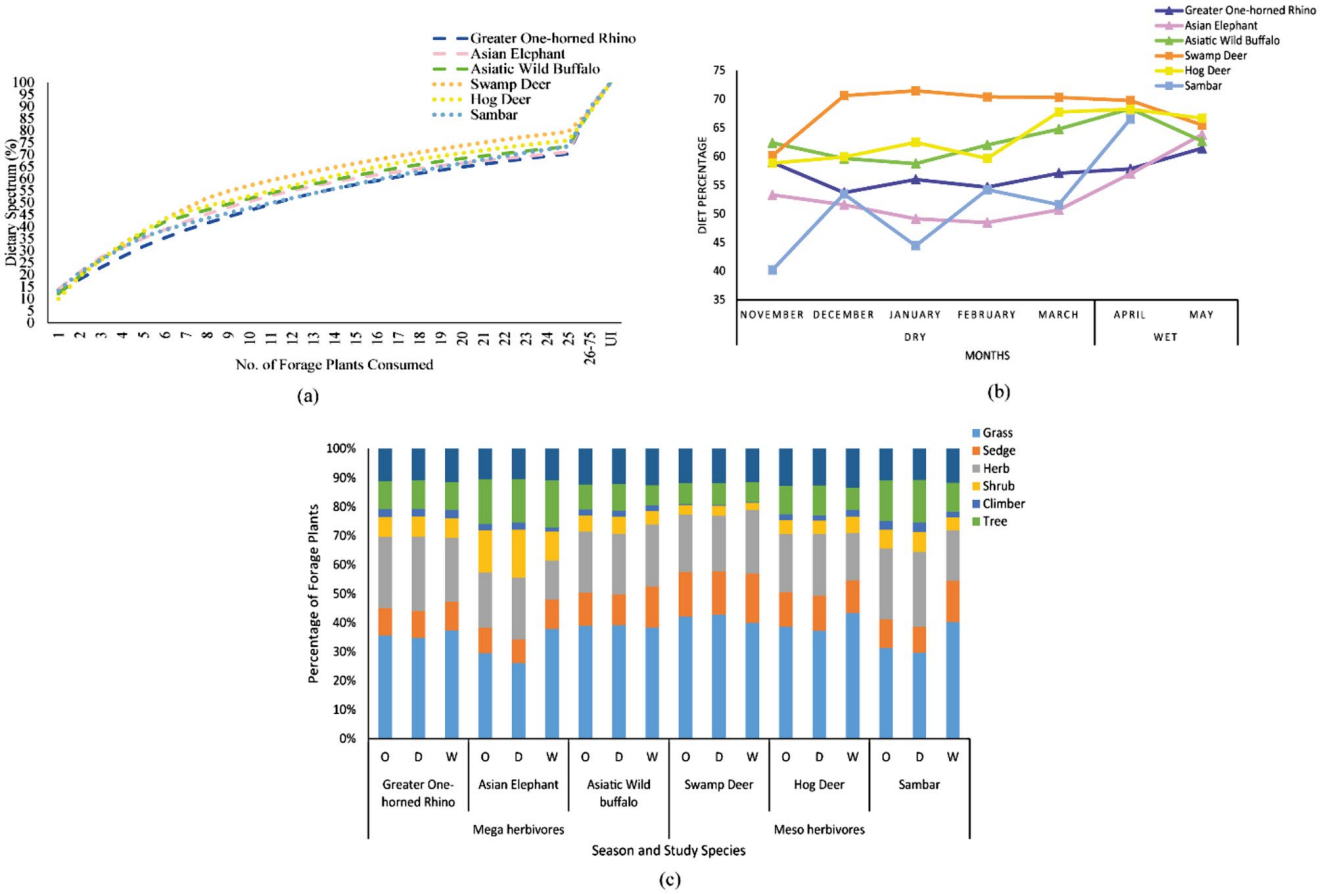
Graph showing (**a**) overall dietary spectrum (%) of major contributing forage plants to the diet, (**b**) monthly consumption of monocots by the six large herbivore species. A high value of the diet percentage suggests a graze-based diet, whereas a low value of the diet percentage suggests a browse-based diet and (**c**) diet composition of mega and meso-herbivores in terms of six growth forms of different forage plants in Kaziranga National Park, Assam.

Throughout the year, 25 major forage plants constituted more than 70% of the diet of large herbivores (Fig. 2a), specifically 79.48% of swamp deer’s, 75.87% of hog deer’s, 73.42% of sambar’s, 73.38% of buffalo’s, 71.04% of elephant’s, and 70.29% of rhino’s diet. The 22 principal forage plant species, namely *Saccharum* spp*.*, *Echinochloa* *crus-galli*, *Cynodon* *dactylon*, *Ziziphus* *jujuba*, *Hemarthria* *compressa*, *Alpinia* *nigra*, *Carex* *vesicaria*, *Kyllinga* *brevifolia*, *Mallotus* *nudiflorus*, *Lippia alba*, *Fimbristylis* *aestivalis*, *Amaranthus spinosus*, *Ageratum conyzoides*, *Duchesnea indica*, *Calamus* *tenuis*, *Oxalis corniculata*, *Imperata* *cylindrica*, *Acmella* *uliginosa*, *Amaranthus viridis*, *Dillenia* *indica*, *Solanum* *americanum*, and *Setaria pumila* contributed more than 2% each. The highest number of identified principal forage species (n = 22) were recorded for hog deer (n = 14; 61.15%) followed by rhino (n = 13; 53.89%), elephant (n = 12; 55.00%), sambar (n = 12; 51.90%), buffalo (n = 11; 53.97%), and swamp deer (n = 11; 59.29%).

### Diet comparison

**Figure 3:**
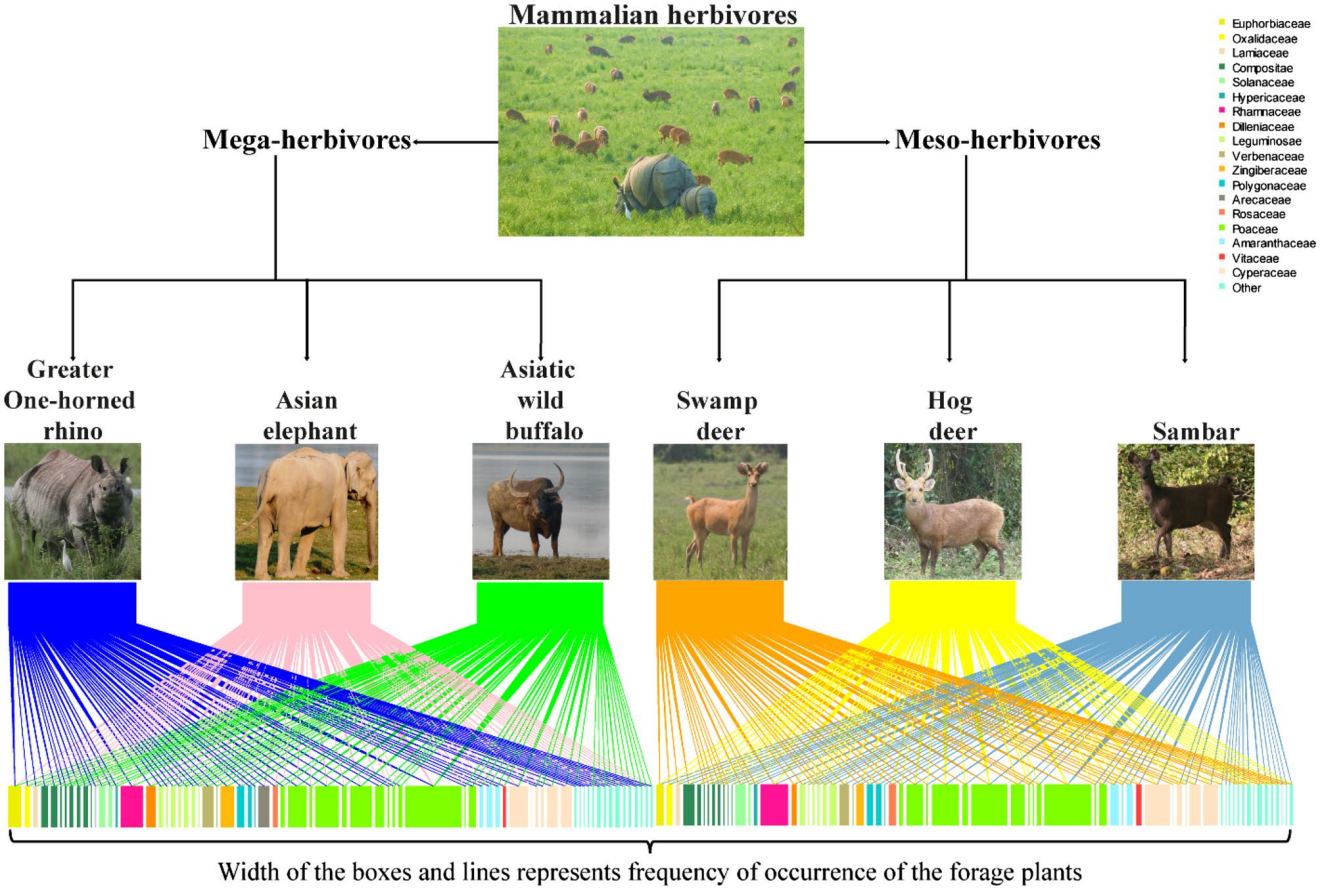
Flow diagram representing the overall bipartite ecological network, which illustrates the diet composition of mega and meso-herbivores. Study species (upper boxes) connected by lines to forage plants (lower boxes) are coloured by family. The width of the lines and lower boxes represent the frequency of occurrence of the forage plants and their respective families in the diet of mega and meso-herbivores in Kaziranga National Park, Assam.

**Figure 4:**
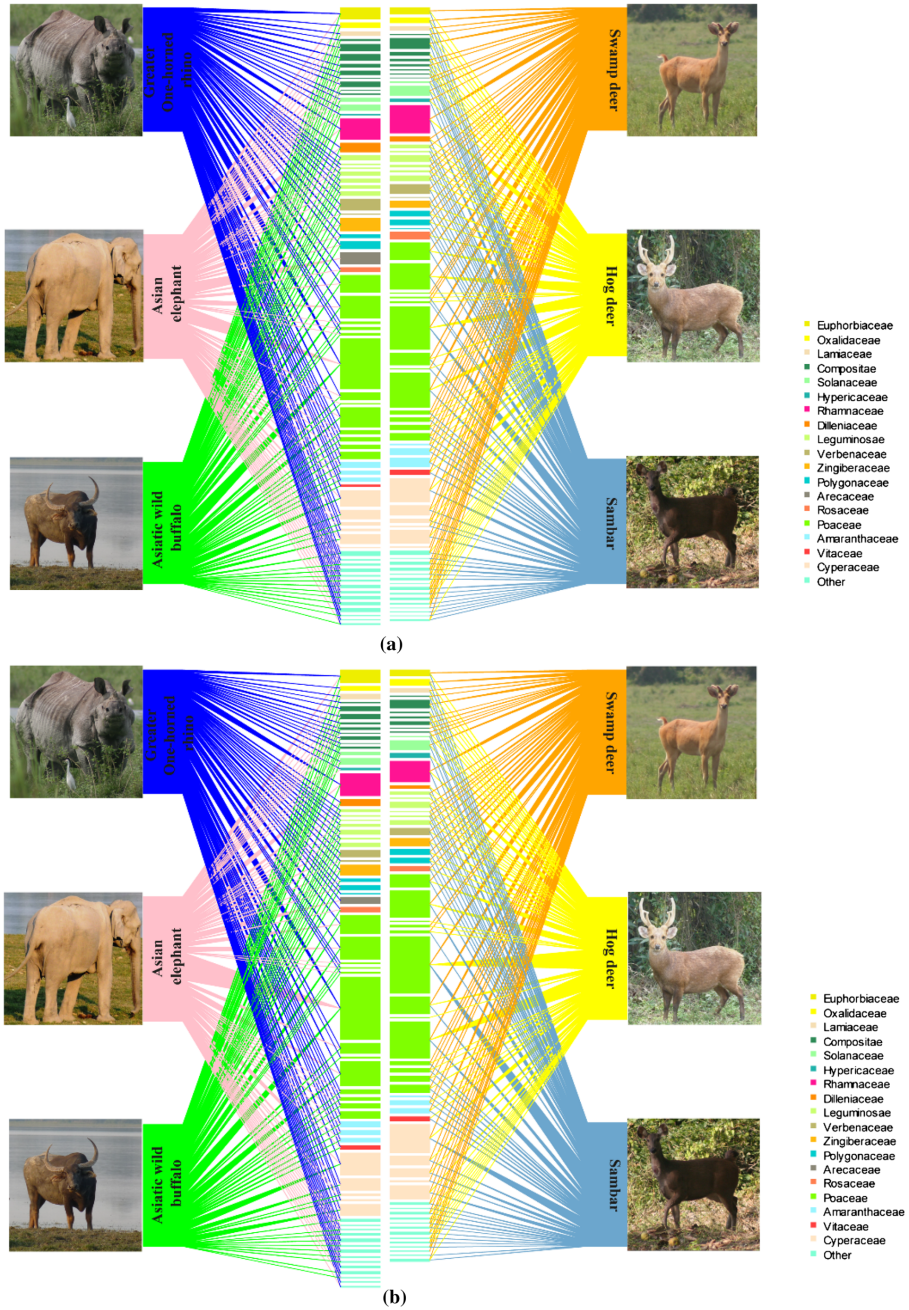
The bipartite ecological network illustrates the diet composition of mega and meso-herbivores. Study species (upper boxes) connected by lines to forage plants (lower boxes) are coloured by family. The width of the lines and lower boxes represent the frequency of occurrence of the forage plants and their respective families in the diet of mega and meso-herbivores in (**a**) dry and (**b**) wet season in Kaziranga National Park, Assam.

The mega and meso-herbivores consumed more monocots during the wet season than the dry season (Fig. 2b). There were significant seasonal differences in the consumption of monocots & monocots, dicots & dicots, and monocots & dicots among the six large herbivores. Between the mega and meso-herbivores, there were significant seasonal differences in the consumption of dicots & dicots and monocots & dicots, and no significant seasonal difference in the consumption of monocots & monocots. Among the six growth forms, grasses were dominant in the diet of all the six herbivores (Fig. 2c). There were significant seasonal differences in the consumption of grasses and herbs among the six large herbivores, and no significant differences in the consumption of sedges, shrubs, climbers, and trees. Between the mega and meso-herbivores, there were significant seasonal differences in the consumption of grasses and trees, and no significant seasonal differences in the consumption of sedges, herbs, shrubs, and climbers. A total of 31 families of forage plants were identified in the diet of the mega-herbivores and 29 in the diet of the meso-herbivores. Overall, the Bipartite Ecological Network (BEN) shows that the members of the family Poaceae contributed the most to the diet of mega and meso-herbivores (Fig. 3). It also shows that the contribution of forage species belonging to the families Poaceae and Cyperaceae, to the diet of both mega and meso-herbivores, increased from dry (34.33 to 57.70%) to wet season (47.27 to 57.06%) (Fig. 4a,b). *C. tenuis* (family Arecaceae) was consumed mostly by the elephant. BEN further revealed that the mean number of shared forage plants in the diet of both mega and meso-herbivores in the dry and wet seasons were 57.6 and 51, respectively. The tall grass *Saccharum* spp. constituted a major part of the diet of mega and meso-herbivores during the wet season. A significant seasonal difference in the consumption of *Saccharum* spp. was observed among the six large herbivores, whereas no significant difference was observed in the consumption of *Saccharum* spp. between the mega and meso-herbivores during different seasons.

Monocots dominated the diet of both mega and meso-herbivores throughout the year (Table 1). Only elephant and sambar consumed a significantly higher proportion of monocots than dicots in the wet season (Table 2). In contrast, there was no significant seasonal difference in the consumption of monocots & dicots by rhino, buffalo, swamp deer, and hog deer. Compared to the dry season, the six herbivores consumed a significantly higher proportion of monocots and a significantly lower proportion of dicots in the wet season.

**Table 1:** Identified and unidentified fragments and forage plants recorded in the diet of mega and meso-herbivores throughout the year during 2013–15 in Kaziranga National Park, Assam. *I* identified, *UI* unidentified, *M* monocots, *D* Dicots.

Study species	Fragments									Forage plants				
	I	I %	UI	UI %	Total	M	M%	D	D%	I	I %	UI	UI %	Total
Greater One-horned Rhino	7519	88.99	930	11.01	8449	4827	57.13	3622	42.87	69	88.99	55	11.01	124
Asian Elephant	7184	89.55	838	10.45	8022	4295	53.54	3727	46.46	67	89.55	38	10.45	105
Asiatic Wild Buffalo	6403	87.77	892	12.23	7295	4567	62.60	2728	37.40	62	87.77	34	12.23	96
Swamp Deer	6595	88.23	880	11.77	7475	5111	68.37	2364	31.63	59	88.23	31	11.77	90
Hog Deer	6419	87.23	940	12.77	7359	4628	62.89	2731	37.11	59	87.23	32	12.77	91
Sambar	6277	89.19	761	10.81	7038	3622	51.46	3416	48.54	63	89.19	29	10.81	92

**Table 2:** Chi-square test for food choices of the mega and meso-herbivores between dry and wet season (M-monocots; D-dicots) during 2013–15 in Kaziranga National Park, Assam.

Forage category	Six large herbivores			Mega and meso-herbivore			Greater one-horned rhino			Asian elephant			Asiatic wild buffalo			Swamp deer			Hog deer			Sambar		
	χ^2^	df	p	χ^2^	df	p	χ^2^	df	p	χ^2^	df	p	χ^2^	df	p	χ^2^	df	p	χ^2^	df	p	χ^2^	df	p
M & M	11.29	5	0.05	3.19	1	0.07	65.60	1	0.00	46.59	1	0.00	71.36	1	0.00	88.78	1	0.00	68.19	1	0.00	99.11	1	0.00
D & D	31.21	5	0.00	4.69	1	0.03	63.48	1	0.00	86.58	1	0.00	57.97	1	0.00	39.80	1	0.00	62.11	1	0.00	173.97	1	0.00
M & D	56.63	5	0.00	6.59	1	0.01	0.64	1	0.43	5.63	1	0.02	1.03	1	0.31	0.08	1	0.78	2.00	1	0.16	11.06	1	0.00
Grass	14.81	5	0.01	4.18	1	0.04	37.64	1	0.00	14.29	1	0.00	47.88	1	0.00	68.79	1	0.00	35.64	1	0.00	62.04	1	0.00
Herb	25.28	5	0.00	0.23	1	0.63	10.88	1	0.00	7.23	1	0.01	8.45	1	0.00	16.64	1	0.00	19.05	1	0.00	16.29	1	0.00
Tree	10.45	5	0.06	4.56	1	0.03	47.02	1	0.00	50.29	1	0.00	24.97	1	0.00	13.26	1	0.00	39.68	1	0.00	85.92	1	0.00
Sedge	2.44	5	0.79	1.34	1	0.25	8.33	1	0.00	37.69	1	0.00	15.51	1	0.00	4.55	1	0.03	3.46	1	0.06	21.56	1	0.00
Shrub	6.79	5	2.37	0.06	1	0.81	3.56	1	0.06	9.00	1	0.00	3.27	1	0.07	2.78	1	0.10	1.14	1	0.29	10.89	1	0.00
Climber	4.30	5	0.51	0.22	1	0.64	10.24	1	0.00	15.71	1	0.00	15.00	1	0.00	9.00	1	0.00	20.43	1	0.00	47.82	1	0.00
*Saccharum* spp^a^	16.62	5	0.01	1.61	1	0.21	–	–	–	–	–	–	–	–	–	–	–	–	–	–	–	–	–	–

^a^Principal forage of mega and meso-herbivores.

Grasses dominated the diet of both mega and meso-herbivores throughout the year (Fig. 2c). Between the dry and wet seasons, the six herbivores consumed significantly different proportions of grasses, herbs, climbers, and trees; and only elephant and sambar consumed a significantly different proportion of shrubs. Excluding hog deer, the study species consumed significantly different proportions of sedges (Table 2).

Among the monocots, *Saccharum* spp., *E. crus-galli*, *C. dactylon*, *H. compressa,* and *A. nigra* contributed the most to the rhino diet, while among the dicots, *Z. jujuba*, *M. nudiflorus*, *L. alba*, *A. spinosus*, and *A. conyzoides* contributed the most (Supplementary Table S1). In the elephant diet, *Saccharum* spp., *C. tenuis*, *C. vesicaria*, *E. crus-galli*, and *H. compressa* contributed the most among the monocots, and *Z. jujuba*, *M. nudiflorus*, *D. indica*, *L. alba*, and *A. conyzoides* contributed the most among the dicots (Supplementary Table S2). In the buffalo diet, *Saccharum* spp., *H. compressa*, *E. crus-galli*, *C. vesicaria*, and *C. dactylon* contributed the most among the monocots and, *Z. jujuba*, *O. corniculata*, *L. alba*, *S. americanum*, and *M. nudiflorus* contributed the most among the dicots (Supplementary Table S3). In the diet of swamp deer, among the monocots, *H. compressa*, *Saccharum* spp., *E. crus-galli*, *C. vesicaria*, and *I. cylindrica* contributed the most, and among the dicots, *Z. jujuba*, *A. uliginosa*, *L. alba*, *S. americanum*, and *O. corniculata* contributed the most (Supplementary Table S4). In the hog deer diet, *H. compressa*, *Saccharum* spp., *E. crus-galli*, *C. vesicaria*, and *C. dactylon* contributed the most among the monocots, and *Z. jujuba*, *A. viridis*, *D. indica*, *S. americanum*, and *L. alba* contributed the most among the dicots (Supplementary Table S5). In the diet of sambar, among the monocots, *Saccharum* spp., *E. crus-galli*, *H. compressa*, *C. vesicaria*, and *I.* *cylindrica* contributed the most, and among the dicots, *Z. jujuba*, *A. uliginosa*, *S. americanum*, *D. indica*, and *M. nudiflorus* contributed the most (Supplementary Table S6).

### Forage quality

Throughout the year, the highest crude protein (CP) was recorded for *E. crus-galli* (12.16%) and lowest for *Saccharum* spp. (6.02%), among the monocots. In the dry season, the highest CP was recorded for *C. tenuis* (10.87%) and lowest for *Saccharum* spp. (4.75%), whereas in the wet season, the highest CP was recorded for *E. crus-galli* (16.47%) and lowest for *A. nigra* (9.11%) (Supplementary Tables S7–S9). Compared to the dry season, the monocots showed a higher mean concentration of CP, calcium (Ca), magnesium (Mg), sodium (Na), potassium (K), and phosphorous (P), and a lower mean concentration of ash content (AC), acid detergent lignin (ADL), acid detergent fibre (ADF), and neutral detergent fibre (NDF) in the wet season. There were significant differences in AC, CP, ADL, Na, K, and P content (Mann–Whitney, p < 0.05) in monocots between the dry and wet seasons (Supplementary Table S10). The most consumed monocot species in mega and meso-herbivores diet, viz. *Saccharum* spp*.*, showed significant differences in AC, CP, ADL, and K concentrations (Mann–Whitney, p < 0.05), between the dry and wet seasons. The monocots, *A.* *nigra*, *C. vesicaria*, *C. dactylon*, *E. crus-galli*, *H. compressa*, and *I. cylindrica* showed a significant difference in CP concentration (Mann–Whitney, p < 0.05), between the dry and wet seasons. Among the dicots, throughout the year and in both the dry and wet seasons, the highest CP concentration was recorded for *A. viridis* and lowest for *D. indica*. Compared to the dry season, dicots showed a higher mean concentration of ADF, NDF, Mg, Na, K, and P, and a lower mean concentration of CP, ADL, and Ca in the wet season. However, there was no significant difference in the nutrient concentration of dicots between dry and wet seasons. In dicots, the most consumed forage species in the diet of mega and meso-herbivores, viz. *Z. jujuba*, showed a significant difference only in ADL concentration (Mann–Whitney, p < 0.05), between the dry and wet seasons.

Throughout the year and in both the dry and wet seasons, dicots with their high CP and mineral concentrations were more nutritious than monocots. While there were significant changes in the nutritional quality parameters of monocots from the dry to wet season, no significant seasonal changes in the nutritional quality of dicots were recorded (Supplementary Table S10). Throughout the year, there were significant differences in CP, ADL, NDF, Ca, Na, and P concentrations (Mann–Whitney, p < 0.05), between monocots and dicots. In the dry season, there were significant differences in CP, NDF, Ca, Na, and P concentrations (Mann–Whitney, p < 0.05) between monocots and dicots. In the wet season, between monocots and dicots, there were significant differences in Ca, and Na concentrations (Mann–Whitney, p < 0.05). The concentrations of ADL, Na, K, and P (Mann–Whitney, p < 0.05) in monocots and dicots differed significantly between the dry and wet seasons.

**Figure 5:**
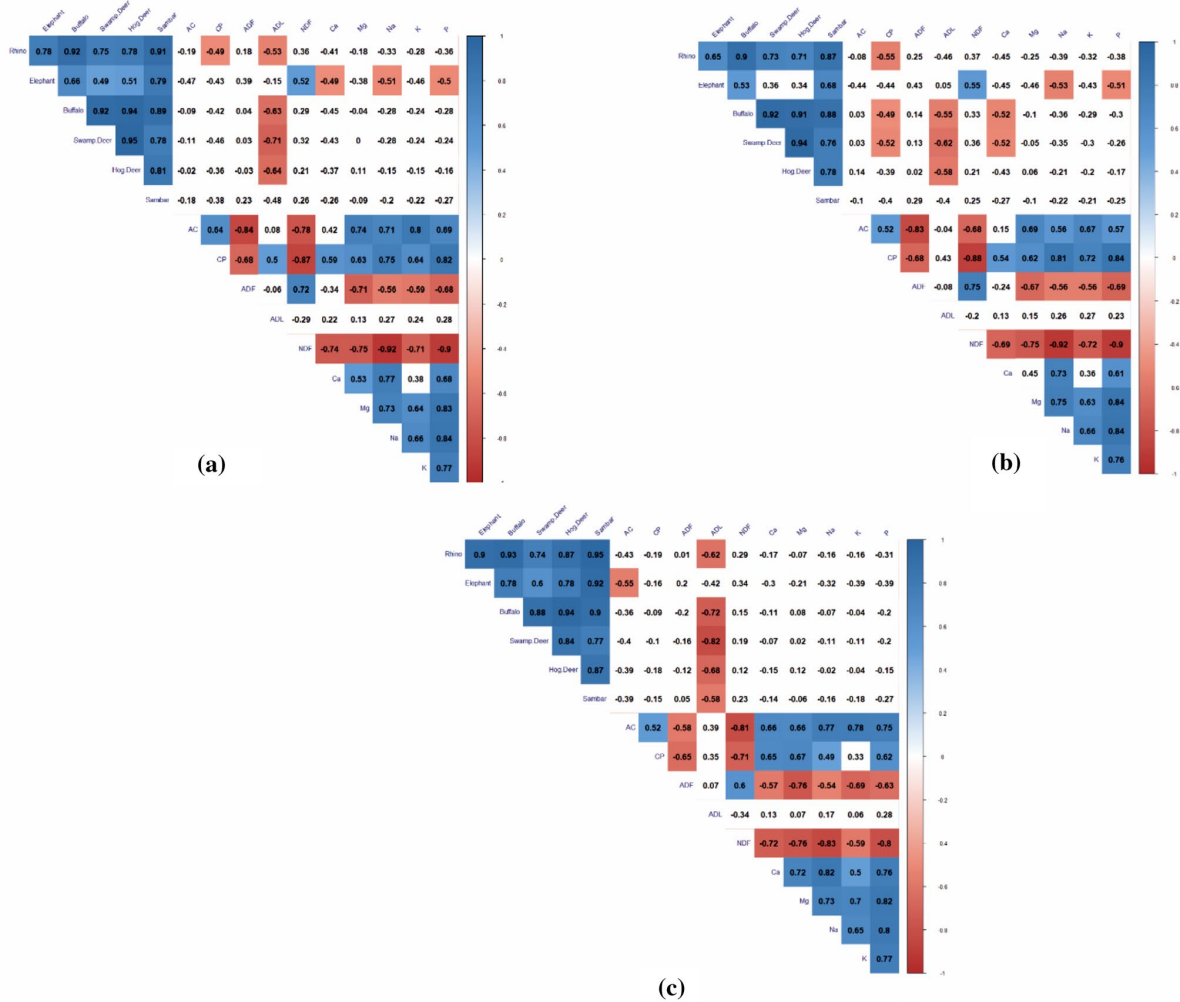
Correlogram showing the relationship between nutrient parameters and the major forage consumed by mega and meso-herbivores in (**a**) overall, (**b**) dry and (**c**) wet season. The values within white boxes represent the insignificant correlation (p > 0.05) whereas the values within red (negative correlation) and blue (positive correlation) boxes represent the significant correlation (p < 0.05).

The top model selection for forage consumption revealed that throughout the year and in the dry season, ADL concentration influenced forage use by mega and meso-herbivores, excluding elephant, whose forage use was influenced by NDF concentration (Supplementary Table S11). In the wet season, AC and ADL concentrations influenced the major forage use. The correlogram revealed that throughout the year, rhino significantly consumed forage with low CP and ADL concentrations; elephant significantly consumed forage rich in NDF and low in Ca, Na, and P; and buffalo, swamp deer, and hog deer significantly consumed forage with low ADL content (Fig. 5a). In the dry season, rhino significantly consumed forage with low CP; elephant significantly consumed forage rich in NDF and low in Na and P; buffalo and swamp deer significantly consumed forage with low CP, ADL, and Ca; and hog deer significantly consumed forage with low ADL concentration (Fig. 5b). In the wet season, rhino, buffalo, swamp deer, hog deer, and sambar significantly consumed forage with low ADL concentration, whereas elephant significantly consumed forage with low AC (Fig. 5c).

## Discussion

With their large body size, the rhino and elephant require almost 150 kg and 240 kg of fodder, respectively, every day, and thus spend most of their time foraging^41,49,52^. Mega and meso-herbivores consumed more monocots in the wet season than in the dry season, although the swamp deer consistently consumed more monocots than other herbivores species. An increase in graze from the dry to the wet season has been recorded in different studies, which also reported 22 to 283 forage plants in the diet of rhino^18,44^, 46 to 112 forage plants in the diet of elephant^18,53,54^, 183 forage plants in the diet of buffalo^48^, 13 to 42 forage plants in the diet of swamp deer^2,29,50^, and 15 to 20 forage plants in the diet of hog deer^29,55^. The diet of sambar is flexible and changes according to the availability of forage^56,57^ and studies have recorded 15 to 180 forage plants in the diet of sambar^50,58^.

In the present study, 124 forage plants in the diet of rhino were recorded of which 69 were identified up to species level and 55 were identified as monocots and dicots. For the elephant, 105 forage plants were recorded in the diet, of which 67 were identified up to species and 38 only as monocots and dicots. For buffalo, 96 forage plants were recorded in diet, of which 62 were identified up to species and 34 only as monocots and dicots. For swamp deer, 90 forage plants were recorded in the diet, of which 59 were identified up to species and 31 only as monocots and dicots. For hog deer, 91 forage plants were recorded in the diet, of which 59 were identified up to species and 32 were only identified as monocots and dicots. For sambar, 92 forage plants were recorded in the diet, of which 63 were identified up to species and remaining 29 only as monocots and dicots. BEN shows that in both the dry and wet seasons, graminoids constituted 50% or more of the diet of both mega and meso-herbivores and Poaceae and Cyperaceae as the most recorded families. In the family Poaceae, the tall grass *Saccharum* spp. was dominant, which might be because of its availability throughout the year. The diet of swamp deer and hog deer had more of the short grass species *H. compressa* in the dry season and more of the tall grass species *Saccharum* spp. in the wet season. *C. tenuis* (family Arecaceae) was mostly consumed by elephant, the possible reason for this could be their ability to exploit resources that are not accessible to other species studied in the area because of its trunk.

We could not detect the presence of important fodder species such as *Mallotus philippinensis* in the diet of any of our study species, even though they were common in the study area and the evidence of browsing on it was observed. In other studies, in similar habitats, its presence could not be detected in the diet of elephant, swamp deer, and hog deer through faecal analysis (e.g., Pradhan et al.^18^, Wegge et al.^29^, and Steinheim et al.^59^). This is an inherent problem in the feeding habit study using micro-histological methods. Further, we failed to detect the presence of *Bombax ceiba*, another common species in the area, though elephants eat its bark. In several other studies (e.g., Brahmachary et al.^45^, and Patar^47^), it was concluded that the large herbivores do not eat *B. ceiba* leaves but debarking, particularly by elephants, is a common feature. A major proportion of the unidentified dicot forage in the diet thus can be attributed to woodland species like *M. philippinensis*, *Terminalia* spp., *Syzygium fruticosum, Mangifera indica*, and *Ficus* spp. These are the reported forage species for rhino and elephant^18,29,41,43,59^.

Experimental studies conducted in North America and Europe showed that mega-herbivores meet their physiological needs by feeding more on dominant species. In high productive ecosystems, this favors plant diversity, whereas in low productive ecosystems, this negatively affects plant diversity^60^. In contrast, selective feeding by meso-herbivores negatively affects plant diversity^61^. Therefore, feeding choices, population, and physiological demand of mega and meso-herbivores can alter the vegetation composition^62^. The present study revealed that throughout the year, among dicot plants, *Z. jujuba* was mostly consumed by both mega and meso-herbivores. Similarly, among monocot plants, *Saccharum* spp. was largely consumed by rhino, elephant, buffalo, and sambar throughout the year. Whereas, *H. compressa* was largely consumed by swamp deer, and hog deer. This suggests the dependence of mega and meso-herbivores on these particular dicots and monocots. The nutrient analysis of major forage also revealed that the major forage species consumed throughout the year by mega and meso-herbivores were more nutritious in the wet season. In the future, any changes in the availability, accessibility, and nutrient content of the major forage plants might affect the population of these mega and meso-herbivores. Therefore, further experimental studies explaining the factors responsible for feeding choices as well as the impact of mega and meso-herbivores on plant vegetation are required, to understand the community vegetation dynamics in KNP.

## Conclusions and conservation implications

The information on the diet composition provides insight into the feeding habits of the mega and meso-herbivores in the wet grasslands of the Brahmaputra floodplains. The findings of this study support the hypothesis that both mega and meso-herbivores consumed a more graze-based diet in the wet season than in the dry season (H_1_). The mega and meso-herbivores grazed more during the wet season, although browse also formed a significant portion of the diet; indicating that both mega and meso-herbivores were grazers and mixed feeders. As monocots were found to be dominant in the forage of rhino, buffalo, swamp deer, and hog deer throughout the year, these species may be more involved in grazing during both the dry and wet seasons. The herbivores, while foraging on nutrient-rich forage, might consume chemically defended forage, resulting in the consumption of toxic plant secondary metabolites (tannins and polyphenols). The detoxification of the secondary metabolites requires more energy. Therefore, to avoid toxic plant secondary metabolites, herbivores might feed on low nutrient quality forage^63^. The shifting of elephant and sambar in the wet season from browsing to grazing indicates their flexibility in utilization of the available forage. The availability of green and nutrient-rich forage is due to the higher moisture regime and controlled burning of wet grasslands. This could be the reason why mega and meso-herbivores feed more on monocots in the wet season^18^. The present study also supports the hypothesis of more nutritious principal monocot forage in the wet season (H_2_). Compared to other herbivores, the diet of rhino, elephant, and sambar consisted more of browse. This contradicts the Jarman–Bell principle, according to which large-bodied herbivores feed mostly on less nutritious graminoids. The seasonal changes in forage availability, mouth size, gut physiology, and predation risk might be responsible for the differences in the forage consumption among mega and meso-herbivores as observed in other studies (e.g., Pradhan et al.^18^, Wegge et al.^29^, Steinheim et al.^59^).

The study suggests that tall and short grasses play a crucial role in meeting the dietary requirements of both mega and meso-herbivores and the importance of riverine alluvial floodplain grasslands in conserving the mega and meso-herbivores. In KNP, the ongoing processes of succession and invasion threaten the grasslands, which in the future might affect the availability of the principal forage plants consumed by mega and meso-herbivores. Thus, grassland-based effective management interventions for conserving the crucial habitat of mega and meso-herbivores are suggested. In the climate crisis and habitat degradation era, the present study will help Park managers to formulate effective conservation strategies for conserving mega and meso-herbivores.

## Materials and methods

### Study area

KNP, in the north-eastern Indian state of Assam, is situated in the floodplains of the Brahmaputra River, which runs along the northern boundary of the Park; and the Karbi Anglong Hills form the southern boundary. KNP, with an area of 429.93 km^2^, lies between latitudes 26° 34′ N and 26° 46′ N and longitudes 93° 08′ E and 93° 36′ E (Fig. 1). An effort to conserve the rhino started with the declaration of Kaziranga as a Reserve Forest in 1908. It was later declared a Wildlife Sanctuary in 1950 and upgraded to a National Park in 1974. Subsequently, it was declared a UNESCO World Heritage Site in 1985 and a Tiger Reserve in 2007^64^. After more than 100 years of conservation efforts, the population of the wildlife in the area has increased^64^. KNP, with its flat terrain and rich alluvial soil, is characterized by numerous permanent water bodies, locally known as *beels*. The climate is of the typical subtropical monsoon type. The total annual precipitation in the study area varies from 1592.8 to 2247.8 mm (2011–2015, Assam Forest Department) with a mean annual precipitation at 1802.7 ± 118.5 mm.

Floods from the Brahmaputra River play a crucial role in the maintenance of the wet grassland ecosystem in KNP, which largely constitutes of tall grasses, short grasses, wetlands, and semi-evergreen forests^65^, and supports one of the world’s largest population of rhino and buffalo, and significant populations of the Eastern swamp deer and elephant^64^. KNP is home to eight mega and meso-herbivores, viz*. R. unicornis*, *E. maximus*, *B. arnee*, *Bos gaurus*, *R. duvaucelii*, *A. porcinus*, *R. unicolor,* and *Muntiacus muntjak*^66^.

### Food habit study

**Figure 6:**
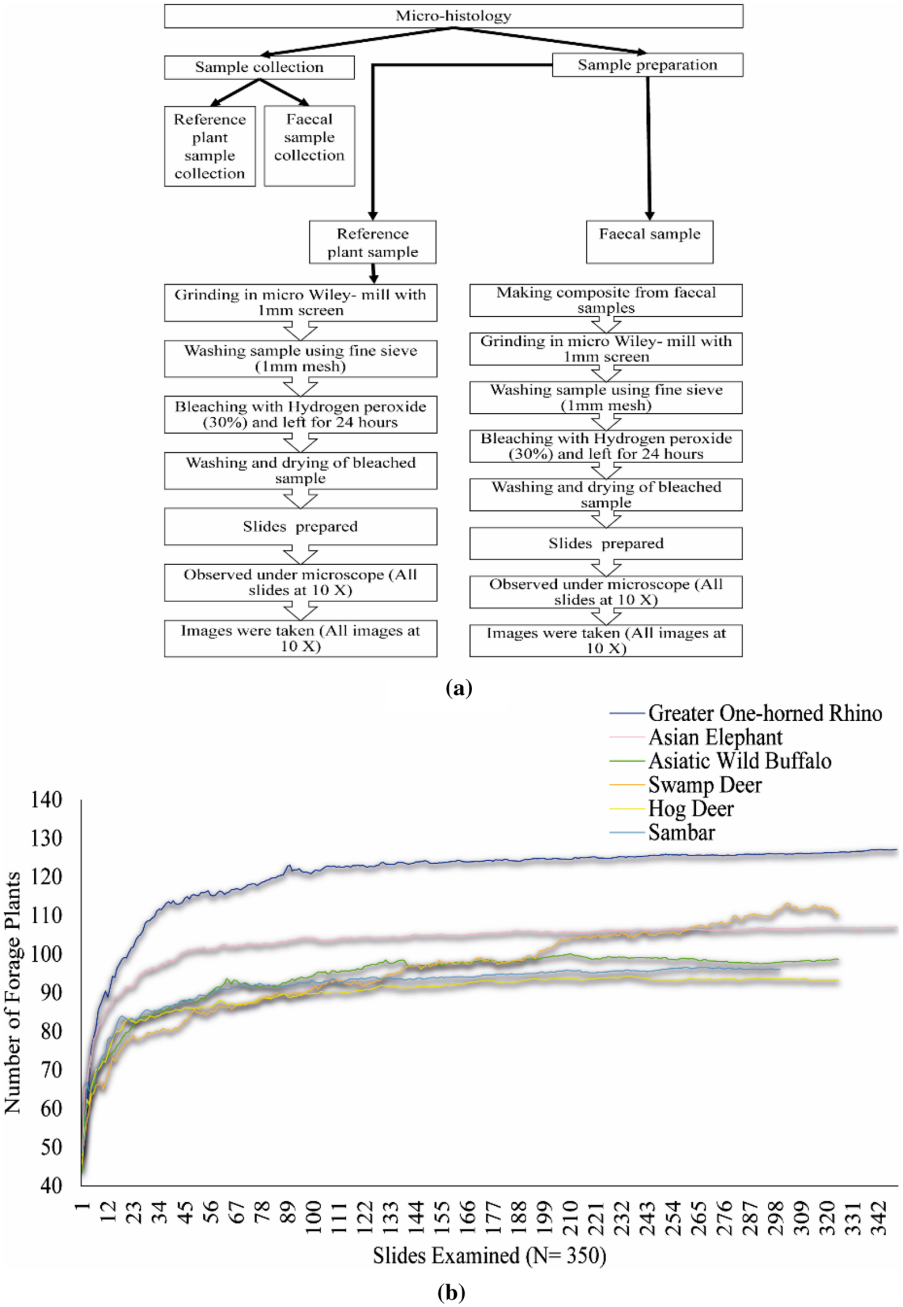
Graph showing (**a**) pathway for micro-histological analysis of plant reference samples and faecal samples and (**b**) overall species accumulation curve for mega and meso-herbivores (Greater one-horned rhino and Asian elephant: N = 350; Asiatic wild buffalo, swamp deer and hog deer: N = 325; sambar: N = 300).

The micro-histological analysis was used to study the feeding habits of the mega and meso-herbivores^2,29,55,67^. This technique involves the preparation of reference slides from the plant material (leaf, stems, flower, and fruit) and comparing it with the slides prepared from the known faecal samples of mega and meso-herbivores^29,68^. The microscopic identification of forage plant fragments was carried out using the keys from Satkopan^69^ and Johnson et al.^70^. For reference samples, 75 potential forage plants were collected from the field, based on literature review and direct field observations^2,29,67^ (Supplementary Table S12). The taxonomic identification of reference plant materials was based on flora of the Kaziranga and Manas National Parks^71–73^. The samples were oven-dried at 60 °C for 48 h^74^, stored in labelled paper bags, and brought to the headquarter for laboratory analysis. The reference samples were processed using the micro-histological technique^29^ in the laboratory (Fig. 6a).

Seventy-five potential forage plants belonging to 31 families were collected. Twenty-eight percent (n = 21) of these were monocots, and 72% (n = 54) were dicots. Among the six growth forms, 17.33% (n = 13) were grasses, 8% (n = 6) were sedges, 6.66% (n = 5) were climbers, 44% (n = 33) were herbs, 17.33% (n = 13) were shrubs, and 6.66% (n = 5) were trees. Of these 75 forage plants, 42.67% (n = 32) were collected from short grasslands, 29.33% (n = 22) from tall grasslands and 28% (n = 21) from woodlands (Supplementary Table S12).

Fresh dung samples of mega-herbivores, viz*.* rhino, elephant and buffalo, and pellets of the meso-herbivores, viz*.* swamp deer, hog deer, and sambar, were collected each month from November 2013 to April 2015 (excluding the flood period from June till October, during which Park remains closed and sample collection was not possible)^64,75^. For the elephant, buffalo, swamp deer, hog deer, and sambar, the faecal samples were collected opportunistically, whereas, for rhino, the faecal samples were collected from latrine sites^76^. These faecal samples were collected from random locations in the short and tall grasslands and woodlands within three forest ranges of KNP, namely Kohora (central), Agoratoli (eastern), and Bagori (western) (Supplementary Table S13). Overall, 1975 faecal samples were collected for both mega and meso-herbivores, of which 1500 samples were collected in the dry season (from November to March) and 475 samples in the wet season (April to May) (Supplementary Table S14). For mega-herbivores, a fresh dung sample, weighing about 400 gm was collected^29,59^. Based on the study by Jachmann and Bell^77^, which showed a positive linear relationship between elephant size, and weight and circumference of boli (individual faeces), we used boli as a guideline for elephant dung sample collection to ensure representation from varied body size individuals^59^. The faecal samples collected from randomly selected habitats within similar locations and ranges were used to make composite samples. For mega-herbivores, five dung samples collected from the same location on the same date were selected randomly and mixed thoroughly to make one composite sample. From this composite sample, 25 g of grounded dung sample was used for micro-histological analysis following Wegge et al.^29^. Similarly, five faecal pellets of the meso-herbivores from each five randomly collected pellet samples were pooled together to make one composite sample. The composite samples were processed using the micro-histological technique following Wegge et al.^29^

Five slides were prepared from each composite sample. Seventy composite samples and 350 slides each were prepared (no. of observations, n = 3500) for rhino and elephant. Sixty-five composite samples and 325 slides each were prepared for buffalo, swamp deer, and hog deer (n = 3250), and 60 composite samples and 300 slides were prepared for sambar (n = 3000). Observations with at least two identifiable fragments were considered for the detection of forage plant species consumed. Whenever possible, identification up to species level was attempted by comparing each sample with the reference plant samples that included leaf, stems, flower, and fruit. We have taken only leaves, stems, flower, and fruit for the preparation of reference materials, and not the bark or roots. Fragments with identifiable features where identification up to species or genus level was difficult due to damaged fragments were categorized as unidentified monocots (including *Bambusa* spp.), and unidentified dicots (including *M. philippinensis*, *Terminalia* spp., and *S. fruticosum*)^18^.

The species accumulation curve was asymptotic at a sample size below the number of slides examined, indicating a sufficient sample size (Fig. 6b). Overall, rhino utilized the maximum number of forage species (n = 124, including both identified and unidentified plants), followed by elephant (n = 105), buffalo (n = 96), sambar (n = 92), hog deer (n = 91), and swamp deer (n = 90) (Table 1).

### Forage quality

Based on the results from the micro-histological analysis, the forage plants consumed in the highest proportion by mega and meso-herbivores were collected twice every month from November 2015 to May 2016. These samples were oven-dried at 60 °C for 48 h in the field and finely ground in 1 mm mesh screen of a Cyclotech’s micro–Wiley mill and stored in airtight plastic bags for estimation of CP, AC, fibre (NDF, ADF, and ADL), and minerals (Ca, P, Mg, K, and Na). Standard protocols were followed to estimate nutrient content (Supplementary Table S15). For Ca and Mg estimation, AAnalyst 700 Atomic Absorption Spectrometer was used with MERCK Certipur Single-Element Standards of Ca and Mg. Similarly, for P estimation, the SMART Spectro 2 Spectrophotometer was used with standard phosphate solution (KH_2_PO_4_)^78^.

### Data analysis

To determine the diet composition of mega and meso-herbivores more precisely, the forage plants identified were categorized further on the basis of (1) graze-to-browse ratio (on monocot and dicot consumption), (2) growth form (grass, sedge, herb, shrub, climber and tree), (3) family, and (4) species. The percentage occurrence of each forage type (graze-to-browse, growth form, family, and species) in the diet of mega and meso-herbivores was determined using the equation of Sparks and Malechek^68^ and Tuboi and Hussain^55^:

$$\mathrm{\% \; contribution \; of \; forage \; plants }= \frac{Number \; of \; identifiable \; fragments \; of \; each \; category}{Total \; number \; of \; identifiable \; fragments \; of \; all \; plant \; species}\times 100.$$

A species accumulation curve was plotted to determine the sampling effort required to adequately examine the diet composition of mega and meso-herbivores. EstimateS version 9 with a 95% confidence interval was used to produce the species accumulation curve^79^. The number of slides examined and the forage plants identified from the faecal samples of the mega and meso-herbivores were plotted in the species accumulation curve^80^. The dietary spectrum of the mega and meso-herbivores was obtained to visualize the pattern of forage utilization on the basis of the major contributing plants. The chi-square test of association and Fisher’s Exact Test were carried out to identify seasonal differences in the forage consumed between the mega and meso-herbivores and among the six herbivores. The tests were performed using SPSS version 22. BEN was used to visualize the forage utilization by mega and meso-herbivores, using R package “bipartite” version 2.11^81^. The forage plants were grouped by family, and only the top 20 abundant families, contributing more than 80% to the diet of mega and meso-herbivores, were highlighted and the rest of the families were grouped under the other category.

The seasonal differences in the nutrient content of major monocot and dicot forage plants were analyzed using a non-parametric Mann–Whitney *U*-test in SPSS version 22. The effect of nutrient factor (predictor) on the use of major forage plants (response) was modelled using a generalized linear model (GLM) and Pearson’s correlation analysis. For GLM, R package “MuMin” vers.1.43.17 and for correlogram, R package “Hmisc” vers. 4.4-0 and “corrplot” vers. 0.84 were used^82–84^.

### Statement for handling plants/plant materials

Experimental research and field studies including collection of plant/plant material for this study, is compliant with the relevant institutional, national, and international guidelines and legislations. The biological samples examined were collected with the permission from the Principal Chief Conservator of Forest (Wildlife) & Chief Wildlife Warden, Government of Assam under section 12 of the Indian Wild Life (Protection) Act, 1972 in O.O No. 868 dated 20th August, 2013. A permission was also obtained from the Director, Kaziranga National Park, Assam in KNP/FG647WII/Research dated 31st October, 2013.

### Ethics approval

Experimental research and field studies including collection of plant/plant material for this study, is compliant with the relevant institutional, national, and international guidelines and legislations.

## Supplementary Information

Supplementary Information.
