# Supplementary material for: Seasonal pattern of food habits of large herbivores in riverine alluvial grasslands of Brahmaputra floodplains, Assam
Source: Sci Rep. 2022 Jan 10;12:482. doi: 10.1038/s41598-021-04295-4 (PMC8748768; doi:10.1038/s41598-021-04295-4)
Supplement: Supplementary file 1 — Supplementary Information. [file 41598_2021_4295_MOESM1_ESM.pdf]

Seasonal pattern of food habits of large herbivores in riverine alluvial grasslands of Brahmaputra floodplains, Assam

Anita Devi[1], Syed Ainul Hussain[1*], Monika Sharma[1], Govindan Veeraswami Gopi[1] and Ruchi Badola[1]

[1]Wildlife Institute of India, Post Box # 18, Chandrabani, Dehra Dun 248001, Uttarakhand, India.

[*] **Correspondence Author:** Syed Ainul Hussain, E-mail: ainul.hussain@gmail.com

Supplementary Information

Table S1. Diet composition (percentage of occurrence) of Greater One-horned Rhino (*Rhinoceros unicornis*) during 2013-15 in Kaziranga National Park, Assam.

Table S2. Diet composition (percentage of occurrence) of Asian elephant (*Elephas maximus*) during 2013-15 in Kaziranga National Park, Assam.

Table S3. Diet composition (percentage of occurrence) of Asiatic wild buffalo (*Bubalus arnee*) during 2013-15 in Kaziranga National Park, Assam.

Table S4. Diet composition (percentage of occurrence) of swamp deer (*Rucervus duvaucelii*) during 2013-15 in Kaziranga National Park, Assam.

Table S5. Diet composition (percentage of occurrence) of hog deer (*Axis porcinus*) during 2013-15 in Kaziranga National Park, Assam.

Table S6. Diet composition (percentage of occurrence) of sambar (*Rusa unicolor*) during 2013-15 in Kaziranga National Park, Assam.

Table S7. Overall (throughout the year) nutrient content (percentage) of major forage plants consumed by mega and meso-herbivores during 2013-15 in Kaziranga National Park, Assam.

Table S8. Dry season nutrient content (percentage) of major forage plants consumed by mega and meso-herbivores during 2013-15 in Kaziranga National Park, Assam.

Table S9. Wet season nutrient content (percentage) of major forage plants consumed by mega and meso-herbivores during 2013-15 in Kaziranga National Park, Assam.

Table S10. Seasonal differences in the nutrient content of major forage consumed by mega and meso-herbivores during 2013-15 in Kaziranga National Park, Assam.

Table S11. Model selection of major forage consumed by mega and meso-herbivores during 2013-15 in Kaziranga National Park, Assam.

Table S12. List of forage plant samples collected and analyzed during 2013-15 in Kaziranga National Park, Assam.

Table S13. GPS coordinates of the faecal collection sites visited frequently for faecal sample collection of mega and meso-herbivores during 2013-15 in Kaziranga National Park, Assam.

Table S14. The number of faecal samples collected for mega and meso-herbivores during 2013-15 in Kaziranga National Park, Assam.

Table S15. The methods used for estimation of the nutrient content of major forage utilized by mega and meso-herbivores during 2013-15 in Kaziranga National Park, Assam.

Table S1. Diet composition (percentage of occurrence) of Greater One-horned Rhino during 2013-15 in Kaziranga National Park, Assam.

Greater One-horned Rhino					
**Plant species**	**Monocot/ Dicot**	**Growth form**	**Percentage of occurrence (%)**		
			**Overall (n=7)**	**Season**	
				**Dry (n=5)**	**Wet (n=2)**
Ziziphus jujuba Mill.	Dicot	Tree	4.40	3.91	5.53
Mallotus nudiflorus (L.) Kulju & Welzen	Dicot	Tree	2.69	3.14	1.66
Lippia alba (Mill.) N.E.Br. ex Britton & P.Wilson	Dicot	Shrub	2.60	3.04	1.62
Amaranthus spinosus L.	Dicot	Herb	2.49	2.68	2.05
Ageratum conyzoides (L.) L.	Dicot	Herb	2.09	2.27	1.70
Persicaria hydropiper (L.) Delarbre	Dicot	Herb	1.70	2.06	0.89
Dillenia indica L.	Dicot	Tree	1.61	1.81	1.16
Solanum americanum Mill.	Dicot	Herb	1.48	1.35	1.78
Crotalaria albida Roth	Dicot	Shrub	1.31	1.23	1.51
Acmella uliginosa (Sw.) Cass.	Dicot	Herb	1.28	1.67	0.39
Duchesnea indica (Andrews) Focke	Dicot	Herb	1.17	1.11	1.31
Leucas aspera (Willd.) Link	Dicot	Herb	1.14	1.40	0.54
Mikania micrantha Kunth	Dicot	Climber	1.05	1.28	0.54
Nelsonia canescens (Lam.) Spreng.	Dicot	Herb	0.91	0.97	0.77
Laphangium luteoalbum (L.) Tzvelev	Dicot	Herb	0.91	1.11	0.46
Merremia sp.	Dicot	Climber	0.89	0.92	0.81
Chenopodium album L.	Dicot	Herb	0.84	0.77	1.01
Solanum viarum Dunal	Dicot	Shrub	0.83	0.82	0.85
Flemingia lineata (L.) Aiton	Dicot	Shrub	0.82	0.84	0.77
Melilotus albus Medik.	Dicot	Herb	0.82	0.90	0.62
Acacia sp.	Dicot	Tree	0.82	0.84	0.77
Grangea maderaspatana (L.) Poir.	Dicot	Herb	0.79	0.84	0.70
Pouzolzia zeylanica (L.) Benn. & R. Br.	Dicot	Herb	0.79	0.78	0.81
Heliotropium indicum L.	Dicot	Herb	0.59	0.68	0.39
Oenanthe javanica (Blume) DC.	Dicot	Herb	0.57	0.56	0.58
Oxalis corniculata L.	Dicot	Herb	0.53	0.56	0.46
Tetrastigma dubium (Lawson) Planch.	Dicot	Climber	0.53	0.20	1.28
Ranunculus sceleratus L.	Dicot	Herb	0.49	0.48	0.50
Rungia pectinata (L.) Nees	Dicot	Herb	0.49	0.56	0.31
Amaranthus viridis L.	Dicot	Herb	0.40	0.17	0.93
Polygonum plebeium R.Br.	Dicot	Herb	0.38	0.26	0.66
Cannabis sativa L.	Dicot	Herb	0.36	0.36	0.35
Hypericum sp.	Dicot	Shrub	0.33	0.09	0.89
Litsea salicifolia (Roxburgh ex Nees) Hook. f.	Dicot	Shrub	0.32	0.38	0.19
Artemisia sp.	Dicot	Herb	0.28	0.02	0.89
Dalbergia sissoo DC.	Dicot	Tree	0.27	0.19	0.46
Cotula hemispherica (Roxb.) Raizada	Dicot	Herb	0.19	0.24	0.08
Stachytarpheta indica (L.) Vahl	Dicot	Shrub	0.18	0.17	0.19
Cajanus scarabaeoides (L.) Thouars	Dicot	Climber	0.17	0.17	0.15
Mimosa spp.	Dicot	Shrub	0.14	0.09	0.27
Urena lobata L.	Dicot	Shrub	0.13	0.07	0.27
Cleome spinosa Jacq.	Dicot	Herb	0.12	0.00	0.39
Rorippa indica (L.) Hiern	Dicot	Herb	0.11	0.09	0.15

Greater One-horned Rhino					
**Plant species**	**Monocot/ Dicot**	**Growth form**	**Percentage of occurrence (%)**		
			**Overall (n=7)**	**Season**	
				**Dry (n=5)**	**Wet (n=2)**
Glochidion multiloculare (Rottler ex Willd.) Voigt	Dicot	Shrub	0.09	0.14	0.00
Ludwigia adscendens (L.) H.Hara	Dicot	Herb	0.07	0.05	0.12
Xanthium strumarium L.	Dicot	Herb	0.04	0.03	0.04
Rumex dentatus L.	Dicot	Herb	0.04	0.00	0.12
Flueggea virosa (Roxb. ex Willd.) Royl	Dicot	Shrub	0.02	0.03	0.00
Youngia japonica (L.) DC.	Dicot	Herb	0.01	0.00	0.04
Saccharum spp.	Monocot	Grass	12.59	12.03	13.88
Echinochloa crus-galli (L.) P.Beauv.	Monocot	Grass	5.49	5.61	5.22
Cynodon dactylon (L.) Pers.	Monocot	Grass	4.94	4.95	4.91
Hemarthria compressa (L.f.) R.Br.	Monocot	Grass	4.37	4.15	4.87
Alpinia nigra (Gaertn.) Burtt	Monocot	Herb	3.52	3.75	2.98
Carex vesicaria L.	Monocot	Sedge	3.33	2.85	4.41
Kyllinga brevifolia Rottb.	Monocot	Sedge	2.84	3.14	2.16
Fimbristylis aestivalis Vahl	Monocot	Sedge	2.54	2.47	2.71
Imperata cylindrica (L.) Raeusch.	Monocot	Grass	1.93	1.79	2.24
Chrysopogon aciculatus (Retz.) Trin.	Monocot	Grass	1.61	1.96	0.81
Setaria pumila (Poir.) Roem. & Schult.	Monocot	Grass	1.15	0.96	1.58
Paspalum conjugatum P.J.Bergius	Monocot	Grass	0.97	0.73	1.51
Panicum sp.	Monocot	Grass	0.89	1.16	0.27
Eleusine indica (L.) Gaertn.	Monocot	Grass	0.89	0.82	1.04
Cyrtococcum sp.	Monocot	Grass	0.70	0.70	0.70
Eleocharis acutangula (Roxb.) Schult.	Monocot	Sedge	0.54	0.56	0.50
Fimbristylis dichotoma (L.) Vahl	Monocot	Sedge	0.17	0.20	0.08
*Oplismenus*sp.	Monocot	Grass	0.13	0.07	0.27
Calamus tenuis Roxb.	Monocot	Shrub	0.09	0.00	0.31
Setaria sp.	Monocot	Grass	0.04	0.00	0.12
Unidentified - 7	Dicot	Unidentified	0.46	0.44	0.50
Unidentified - 12	Dicot	Unidentified	0.44	0.51	0.27
Unidentified - 18	Dicot	Unidentified	0.20	0.26	0.08
Unidentified - 33	Dicot	Unidentified	0.19	0.24	0.08
Unidentified - 43	Dicot	Unidentified	0.19	0.15	0.27
Unidentified - 47	Dicot	Unidentified	0.15	0.00	0.50
Unidentified - 32	Dicot	Unidentified	0.12	0.12	0.12
Unidentified - 11	Dicot	Unidentified	0.11	0.12	0.08
Unidentified - 36	Dicot	Unidentified	0.09	0.10	0.08
Unidentified - 10	Dicot	Unidentified	0.07	0.10	0.00
Unidentified - 14	Dicot	Unidentified	0.07	0.10	0.00
Unidentified - 16	Dicot	Unidentified	0.07	0.07	0.08
Unidentified - 22	Dicot	Unidentified	0.07	0.09	0.04
Unidentified - 35	Dicot	Unidentified	0.06	0.03	0.12
Unidentified - 31	Dicot	Unidentified	0.05	0.05	0.04
Unidentified - 4	Dicot	Unidentified	0.04	0.05	0.00
Unidentified - 5	Dicot	Unidentified	0.04	0.03	0.04
Unidentified - 13	Dicot	Unidentified	0.04	0.03	0.04
Unidentified - 19	Dicot	Unidentified	0.04	0.05	0.00
Unidentified - 20	Dicot	Unidentified	0.04	0.05	0.00
Unidentified - 52	Dicot	Unidentified	0.04	0.03	0.04

Greater One-horned Rhino					
Plant species	Monocot/ Dicot	Growth form	Percentage of occurrence (%)		
			Overall (n=7)	Season	
				Dry (n=5)	Wet (n=2)
Unidentified - 23	Dicot	Unidentified	0.02	0.03	0.00
Unidentified - 8	Dicot	Unidentified	0.01	0.02	0.00
Unidentified - 9	Monocot	Unidentified	1.46	1.74	0.81
Unidentified - 15	Monocot	Unidentified	1.20	1.13	1.35
Unidentified - 42	Monocot	Unidentified	0.73	0.80	0.58
Unidentified - 41	Monocot	Unidentified	0.69	0.61	0.85
Unidentified - 38	Monocot	Unidentified	0.51	0.41	0.73
Unidentified - 49	Monocot	Unidentified	0.44	0.31	0.73
Unidentified - 40	Monocot	Unidentified	0.36	0.29	0.50
Unidentified - 39	Monocot	Unidentified	0.33	0.29	0.43
Unidentified - 53	Monocot	Unidentified	0.33	0.24	0.54
Unidentified - 29	Monocot	Unidentified	0.30	0.41	0.04
Unidentified - 50	Monocot	Unidentified	0.27	0.34	0.12
Unidentified - 37	Monocot	Unidentified	0.24	0.19	0.35
Unidentified - 46	Monocot	Unidentified	0.19	0.17	0.23
Unidentified - 48	Monocot	Unidentified	0.17	0.10	0.31
Unidentified - 51	Monocot	Unidentified	0.15	0.03	0.43
Unidentified - 54	Monocot	Unidentified	0.15	0.14	0.19
Unidentified - 44	Monocot	Unidentified	0.14	0.09	0.27
Unidentified - 21	Monocot	Unidentified	0.12	0.15	0.04
Unidentified - 30	Monocot	Unidentified	0.11	0.10	0.12
Unidentified - 45	Monocot	Unidentified	0.11	0.07	0.19
Unidentified - 1	Monocot	Unidentified	0.06	0.07	0.04
Unidentified - 25	Monocot	Unidentified	0.06	0.02	0.15
Unidentified - 26	Monocot	Unidentified	0.06	0.09	0.00
Unidentified - 17	Monocot	Unidentified	0.05	0.05	0.04
Unidentified - 24	Monocot	Unidentified	0.05	0.05	0.04
Unidentified - 34	Monocot	Unidentified	0.05	0.05	0.04
Unidentified - 6	Monocot	Unidentified	0.04	0.05	0.00
Unidentified - 2	Monocot	Unidentified	0.02	0.03	0.00
Unidentified - 28	Monocot	Unidentified	0.02	0.03	0.00
Unidentified - 3	Monocot	Unidentified	0.01	0.02	0.00
Unidentified - 27	Monocot	Unidentified	0.01	0.02	0.00
Unidentified - 55	Monocot	Unidentified	0.01	0.02	0.00
Identified Monocot (%)			48.72	47.90	50.56
Unidentified Monocot (%)			8.42	8.10	9.12
Total Monocot (%)			57.13	56.00	59.68
Identified Dicot (%)			40.28	41.30	37.96
Unidentified Dicot (%)			2.59	2.70	2.36
Total Dicot (%)			42.87	44.00	40.32
Total (%)			100.00	100.00	100.00

n- number of months

Table S2. Diet composition (percentage of occurrence) of Asian elephant (*Elephas maximus*) during 2013-15 in Kaziranga National Park, Assam.

Asian Elephant					
**Plant species**	**Monocot/ Dicot**	**Growth form**	**Percentage of occurrence (%)**		
			**Overall (n=7)**	**Season**	
				**Dry (n=5)**	**Wet (n=2)**
Ziziphus jujuba Mill.	Dicot	Tree	6.18	6.16	6.24
Mallotus nudiflorus (L.) Kulju & Welzen	Dicot	Tree	4.11	3.51	5.59
Dillenia indica L.	Dicot	Tree	3.35	3.62	2.71
Lippia alba (Mill.) N.E.Br. ex Britton & P.Wilson	Dicot	Shrub	2.49	2.70	1.98
Ageratum conyzoides (L.) L.	Dicot	Herb	1.40	1.72	0.60
Acmella uliginosa (Sw.) Cass.	Dicot	Herb	1.31	1.40	1.08
Litsea salicifolia (Roxburgh ex Nees) Hook. f.	Dicot	Shrub	1.27	1.40	0.95
Persicaria hydropiper (L.) Delarbre	Dicot	Herb	1.21	1.46	0.60
Acacia sp.	Dicot	Tree	1.02	0.81	1.55
Polygonum plebeium R.Br.	Dicot	Herb	1.02	1.25	0.47
Grangea maderaspatana (L.) Poir.	Dicot	Herb	0.93	1.11	0.52
Flemingia lineata (L.) Aiton	Dicot	Shrub	0.92	1.11	0.47
Chenopodium album L.	Dicot	Herb	0.90	0.98	0.69
Nelsonia canescens (Lam.) Spreng.	Dicot	Herb	0.89	1.02	0.56
Urena lobata L.	Dicot	Shrub	0.87	0.77	1.12
Ranunculus sceleratus L.	Dicot	Herb	0.81	0.75	0.95
Dalbergia sissoo DC.	Dicot	Tree	0.80	0.98	0.34
Duchesnea indica (Andrews) Focke	Dicot	Herb	0.79	0.82	0.69
Mikania micrantha Kunth	Dicot	Climber	0.76	0.93	0.34
Laphangium luteoalbum (L.) Tzvelev	Dicot	Herb	0.71	0.81	0.47
Cannabis sativa L.	Dicot	Herb	0.71	0.88	0.30
Ludwigia adscendens (L.) H.Hara	Dicot	Herb	0.66	0.65	0.69
Solanum americanum Mill.	Dicot	Herb	0.65	0.67	0.60
Amaranthus viridis L.	Dicot	Herb	0.59	0.58	0.60
Leucas aspera (Willd.) Link	Dicot	Herb	0.56	0.58	0.52
Amaranthus spinosus L.	Dicot	Herb	0.54	0.51	0.60
Solanum viarum Dunal	Dicot	Shrub	0.54	0.54	0.52
Pouzolzia zeylanica (L.) Benn. & R. Br.	Dicot	Herb	0.50	0.42	0.69
Cajanus scarabaeoides (L.) Thouars	Dicot	Climber	0.49	0.51	0.43
Tetrastigma dubium (Lawson) Planch.	Dicot	Climber	0.47	0.56	0.26
Oxalis corniculata L.	Dicot	Herb	0.46	0.49	0.39
Melilotus albus Medik.	Dicot	Herb	0.45	0.46	0.43
Glochidion multiloculare (Rottler ex Willd.) Voigt	Dicot	Shrub	0.45	0.49	0.34
Merremia sp.	Dicot	Climber	0.41	0.47	0.26
Crotalaria albida Roth	Dicot	Shrub	0.41	0.49	0.22
Hypericum sp.	Dicot	Shrub	0.37	0.44	0.22
Rorippa indica (L.) Hiern	Dicot	Herb	0.35	0.47	0.04
Cotula hemispherica (Roxb.) Raizada	Dicot	Herb	0.24	0.33	0.00
Rungia pectinata (L.) Nees	Dicot	Herb	0.22	0.21	0.26
Heliotropium indicum L.	Dicot	Herb	0.20	0.26	0.04
Oenanthe javanica (Blume) DC.	Dicot	Herb	0.16	0.19	0.09
Stachytarpheta indica (L.) Vahl	Dicot	Shrub	0.11	0.12	0.09
Xanthium strumarium L.	Dicot	Herb	0.05	0.05	0.04

Asian Elephant					
Plant species	Monocot/ Dicot	Growth form	Percentage of occurrence (%)		
			Overall (n=7)	Season	
				Dry (n=5)	Wet (n=2)
Mimosa spp.	Dicot	Shrub	0.04	0.05	0.00
Youngia japonica (L.) DC.	Dicot	Herb	0.02	0.04	0.00
Artemisia sp.	Dicot	Herb	0.01	0.02	0.00
Saccharum spp.	Monocot	Grass	13.99	11.69	19.61
Calamus tenuis Roxb.	Monocot	Shrub	7.07	8.29	4.09
Carex vesicaria L.	Monocot	Sedge	3.80	3.05	5.63
Echinochloa crus-galli (L.) P.Beauv.	Monocot	Grass	3.58	3.48	3.83
Hemarthria compressa (L.f.) R.Br.	Monocot	Grass	3.22	2.35	5.33
Alpinia nigra (Gaertn.) Burtt	Monocot	Herb	2.75	3.25	1.55
Cynodon dactylon (L.) Pers.	Monocot	Grass	2.33	2.09	2.92
Fimbristylis aestivalis Vahl	Monocot	Sedge	2.12	2.11	2.15
Setaria pumila (Poir.) Roem. & Schult.	Monocot	Grass	1.86	1.93	1.68
Imperata cylindrica (L.) Raeusch.	Monocot	Grass	1.73	1.32	2.75
Kyllinga brevifolia Rottb.	Monocot	Sedge	1.58	1.54	1.68
Eleocharis acutangula (Roxb.) Schult.	Monocot	Sedge	0.85	1.02	0.43
*Oplismenus*sp.	Monocot	Grass	0.75	0.95	0.26
Paspalum conjugatum P.J.Bergius	Monocot	Grass	0.70	0.81	0.43
Eleusine indica (L.) Gaertn.	Monocot	Grass	0.65	0.63	0.69
Chrysopogon aciculatus (Retz.) Trin.	Monocot	Grass	0.42	0.49	0.26
Fimbristylis dichotoma (L.) Vahl	Monocot	Sedge	0.39	0.44	0.26
Cyrtococcum sp.	Monocot	Grass	0.15	0.18	0.09
Setaria sp.	Monocot	Grass	0.11	0.16	0.00
Cyperus squarrosus L.	Monocot	Sedge	0.05	0.07	0.00
Panicum sp.	Monocot	Grass	0.05	0.04	0.09
Unidentified - 21	Dicot	Unidentified	0.87	1.04	0.47
Unidentified - 23	Dicot	Unidentified	0.62	0.53	0.86
Unidentified - 27	Dicot	Unidentified	0.55	0.70	0.17
Unidentified - 22	Dicot	Unidentified	0.49	0.67	0.04
Unidentified - 15	Dicot	Unidentified	0.44	0.42	0.47
Unidentified - 20	Dicot	Unidentified	0.34	0.47	0.00
Unidentified - 10	Dicot	Unidentified	0.31	0.32	0.30
Unidentified - 29	Dicot	Unidentified	0.26	0.33	0.09
Unidentified - 7	Dicot	Unidentified	0.22	0.07	0.60
Unidentified - 11	Dicot	Unidentified	0.17	0.11	0.34
Unidentified - 4	Dicot	Unidentified	0.16	0.18	0.13
Unidentified - 18	Dicot	Unidentified	0.12	0.14	0.09
Unidentified - 8	Dicot	Unidentified	0.11	0.16	0.00
Unidentified - 36	Dicot	Unidentified	0.11	0.04	0.30
Unidentified - 30	Dicot	Unidentified	0.10	0.14	0.00
Unidentified - 26	Dicot	Unidentified	0.06	0.05	0.09
Unidentified - 35	Dicot	Unidentified	0.05	0.04	0.09
Unidentified - 37	Dicot	Unidentified	0.05	0.07	0.00
Unidentified - 6	Monocot	Unidentified	1.20	1.09	1.46
Unidentified - 25	Monocot	Unidentified	0.90	0.56	1.72
Unidentified - 17	Monocot	Unidentified	0.81	0.70	1.08
Unidentified - 31	Monocot	Unidentified	0.55	0.49	0.69
Unidentified - 19	Monocot	Unidentified	0.34	0.30	0.43

Asian Elephant					
Plant species	Monocot/ Dicot	Growth form	Percentage of occurrence (%)		
			Overall (n=7)	Season	
				Dry (n=5)	Wet (n=2)
Unidentified - 1	Monocot	Unidentified	0.21	0.28	0.04
Unidentified - 5	Monocot	Unidentified	0.17	0.18	0.17
Unidentified - 24	Monocot	Unidentified	0.17	0.21	0.09
Unidentified - 2	Monocot	Unidentified	0.15	0.21	0.00
Unidentified - 13	Monocot	Unidentified	0.12	0.16	0.04
Unidentified - 9	Monocot	Unidentified	0.11	0.07	0.22
Unidentified - 12	Monocot	Unidentified	0.10	0.14	0.00
Unidentified - 28	Monocot	Unidentified	0.10	0.12	0.04
Unidentified - 33	Monocot	Unidentified	0.10	0.04	0.26
Unidentified - 3	Monocot	Unidentified	0.09	0.07	0.13
Unidentified - 32	Monocot	Unidentified	0.09	0.09	0.09
Unidentified - 34	Monocot	Unidentified	0.06	0.09	0.00
Unidentified - 38	Monocot	Unidentified	0.06	0.00	0.22
Unidentified - 16	Monocot	Unidentified	0.05	0.05	0.04
Unidentified - 14	Monocot	Unidentified	0.01	0.02	0.00
Identified Monocot (%)			48.14	45.87	53.72
Unidentified Monocot (%)			5.40	4.86	6.71
Total Monocot (%)			53.54	50.73	60.43
Identified Dicot (%)			41.41	43.81	35.53
Unidentified Dicot (%)			5.05	5.46	4.04
Total Dicot (%)			46.46	49.27	39.57
Total (%)			100.00	100.00	100.00

n- number of months

Table S3. Diet composition (percentage of occurrence) of Asiatic wild buffalo (*Bubalus arnee*) during 2013-15 in Kaziranga National Park, Assam.

Asiatic Wild Buffalo					
**Plant species**	**Monocot/ Dicot**	**Growth form**	**Percentage of occurrence (%)**		
			**Overall (n=7)**	**Season**	
				**Dry (n=5)**	**Wet (n=2)**
Ziziphus jujuba Mill.	Dicot	Tree	4.80	5.00	4.10
Oxalis corniculata L.	Dicot	Herb	2.44	2.51	2.20
Lippia alba (Mill.) N.E.Br. ex Britton & P.Wilson	Dicot	Shrub	1.91	2.14	1.10
Solanum americanum Mill.	Dicot	Herb	1.82	1.80	1.89
Mallotus nudiflorus (L.) Kulju & Welzen	Dicot	Tree	1.81	1.87	1.59
Persicaria hydropiper (L.) Delarbre	Dicot	Herb	1.77	1.78	1.71
Acmella uliginosa (Sw.) Cass.	Dicot	Herb	1.62	1.61	1.65
Amaranthus viridis L.	Dicot	Herb	1.40	1.43	1.28
Leucas aspera (Willd.) Link	Dicot	Herb	1.34	1.04	2.38
Solanum viarum Dunal	Dicot	Shrub	1.08	1.17	0.79
Duchesnea indica (Andrews) Focke	Dicot	Herb	1.03	0.99	1.16
Mikania micrantha Kunth	Dicot	Climber	1.03	1.08	0.86
Polygonum plebeium R.Br.	Dicot	Herb	0.96	0.92	1.10
Dillenia indica L.	Dicot	Tree	0.95	1.01	0.73
Flemingia lineata (L.) Aiton	Dicot	Shrub	0.90	1.06	0.37
Nelsonia canescens (Lam.) Spreng.	Dicot	Herb	0.88	1.02	0.37
Crotalaria albida Roth	Dicot	Shrub	0.88	0.78	1.22
Chenopodium album L.	Dicot	Herb	0.86	0.74	1.28
Acacia sp.	Dicot	Tree	0.85	0.97	0.43
Melilotus albus Medik.	Dicot	Herb	0.78	0.81	0.67
Ageratum conyzoides (L.) L.	Dicot	Herb	0.58	0.49	0.86
Amaranthus spinosus L.	Dicot	Herb	0.56	0.49	0.79
Tetrastigma dubium (Lawson) Planch.	Dicot	Climber	0.56	0.46	0.92
Pouzolzia zeylanica (L.) Benn. & R. Br.	Dicot	Herb	0.53	0.58	0.37
Litsea salicifolia (Roxburgh ex Nees) Hook. f.	Dicot	Shrub	0.51	0.51	0.49
Merremia sp.	Dicot	Climber	0.49	0.57	0.24
Grangea maderaspatana (L.) Poir.	Dicot	Herb	0.45	0.55	0.12
Oenanthe javanica (Blume) DC.	Dicot	Herb	0.37	0.37	0.37
Ranunculus sceleratus L.	Dicot	Herb	0.34	0.44	0.00
Laphangium luteoalbum (L.) Tzvelev	Dicot	Herb	0.29	0.35	0.06
Dalbergia sissoo DC.	Dicot	Tree	0.23	0.28	0.06
Rungia pectinata (L.) Nees	Dicot	Herb	0.16	0.19	0.06
Cannabis sativa L.	Dicot	Herb	0.16	0.19	0.06
Cotula hemispherica (Roxb.) Raizada	Dicot	Herb	0.14	0.18	0.00
Urena lobata L.	Dicot	Shrub	0.12	0.05	0.37
Ludwigia adscendens (L.) H.Hara	Dicot	Herb	0.07	0.09	0.00
Stachytarpheta indica (L.) Vahl	Dicot	Shrub	0.07	0.04	0.18
Rorippa indica (L.) Hiern	Dicot	Herb	0.04	0.02	0.12
Youngia japonica (L.) DC.	Dicot	Herb	0.04	0.04	0.06
Hypericum sp.	Dicot	Shrub	0.04	0.05	0.00
Mimosa spp.	Dicot	Shrub	0.04	0.04	0.06
Heliotropium indicum L.	Dicot	Herb	0.03	0.02	0.06
Artemisia sp.	Dicot	Herb	0.01	0.00	0.06

Asiatic Wild Buffalo					
**Plant species**	**Monocot/ Dicot**	**Growth form**	**Percentage of occurrence (%)**		
			**Overall (n=7)**	**Season**	
				**Dry (n=5)**	**Wet (n=2)**
Saccharum spp.	Monocot	Grass	12.05	12.33	11.06
Hemarthria compressa (L.f.) R.Br.	Monocot	Grass	7.35	7.37	7.27
Echinochloa crus-galli (L.) P.Beauv.	Monocot	Grass	6.94	6.94	6.91
Carex vesicaria L.	Monocot	Sedge	5.76	5.73	5.87
Cynodon dactylon (L.) Pers.	Monocot	Grass	5.13	5.16	5.01
Kyllinga brevifolia Rottb.	Monocot	Sedge	2.54	2.10	4.03
Alpinia nigra (Gaertn.) Burtt	Monocot	Herb	2.43	2.35	2.69
Imperata cylindrica (L.) Raeusch.	Monocot	Grass	2.33	2.33	2.32
Fimbristylis aestivalis Vahl	Monocot	Sedge	2.22	1.86	3.48
Setaria pumila (Poir.) Roem. & Schult.	Monocot	Grass	1.58	1.61	1.47
Eleusine indica (L.) Gaertn.	Monocot	Grass	1.12	1.10	1.22
Paspalum conjugatum P.J.Bergius	Monocot	Grass	0.78	0.71	1.04
Fimbristylis dichotoma (L.) Vahl	Monocot	Sedge	0.56	0.53	0.67
Cyrtococcum sp.	Monocot	Grass	0.56	0.62	0.37
Chrysopogon aciculatus (Retz.) Trin.	Monocot	Grass	0.41	0.32	0.73
Eleocharis acutangula (Roxb.) Schult.	Monocot	Sedge	0.34	0.41	0.12
*Oplismenus*sp.	Monocot	Grass	0.30	0.35	0.12
Setaria sp.	Monocot	Grass	0.23	0.12	0.61
Panicum sp.	Monocot	Grass	0.19	0.18	0.24
Unidentified - 13	Dicot	Unidentified	0.47	0.46	0.49
Unidentified - 26	Dicot	Unidentified	0.37	0.46	0.06
Unidentified - 18	Dicot	Unidentified	0.27	0.25	0.37
Unidentified - 9	Dicot	Unidentified	0.26	0.28	0.18
Unidentified - 10	Dicot	Unidentified	0.22	0.27	0.06
Unidentified - 5	Dicot	Unidentified	0.21	0.27	0.00
Unidentified - 19	Dicot	Unidentified	0.21	0.27	0.00
Unidentified - 12	Dicot	Unidentified	0.19	0.19	0.18
Unidentified - 2	Dicot	Unidentified	0.05	0.07	0.00
Unidentified - 14	Dicot	Unidentified	0.05	0.07	0.00
Unidentified - 20	Dicot	Unidentified	0.04	0.05	0.00
Unidentified - 24	Dicot	Unidentified	0.04	0.05	0.00
Unidentified - 31	Dicot	Unidentified	0.03	0.04	0.00
Unidentified - 22	Dicot	Unidentified	0.01	0.02	0.00
Unidentified - 27	Dicot	Unidentified	0.01	0.00	0.06
Unidentified - 1	Monocot	Unidentified	2.12	2.05	2.38
Unidentified - 6	Monocot	Unidentified	1.48	1.54	1.28
Unidentified - 16	Monocot	Unidentified	1.37	1.25	1.77
Unidentified - 8	Monocot	Unidentified	1.01	1.06	0.86
Unidentified - 4	Monocot	Unidentified	0.77	0.35	2.20
Unidentified - 7	Monocot	Unidentified	0.67	0.72	0.49
Unidentified - 29	Monocot	Unidentified	0.48	0.48	0.49
Unidentified - 3	Monocot	Unidentified	0.45	0.46	0.43
Unidentified - 17	Monocot	Unidentified	0.41	0.34	0.67
Unidentified - 11	Monocot	Unidentified	0.25	0.32	0.00
Unidentified - 15	Monocot	Unidentified	0.22	0.27	0.06
Unidentified - 25	Monocot	Unidentified	0.16	0.14	0.24
Unidentified - 33	Monocot	Unidentified	0.14	0.16	0.06

Asiatic Wild Buffalo					
Plant species	Monocot/ Dicot	Growth form	Percentage of occurrence (%)		
			Overall (n=7)	Season	
				Dry (n=5)	Wet (n=2)
Unidentified - 21	Monocot	Unidentified	0.07	0.07	0.06
Unidentified - 23	Monocot	Unidentified	0.05	0.07	0.00
Unidentified - 28	Monocot	Unidentified	0.04	0.02	0.12
Unidentified - 34	Monocot	Unidentified	0.04	0.05	0.00
Unidentified - 32	Monocot	Unidentified	0.03	0.04	0.00
Unidentified - 30	Monocot	Unidentified	0.01	0.02	0.00
Identified Monocot (%)			52.82	52.11	55.26
Unidentified Monocot (%)			9.79	9.40	11.12
Total Monocot (%)			62.60	61.51	66.38
Identified Dicot (%)			34.96	35.75	32.21
Unidentified Dicot (%)			2.44	2.74	1.41
Total Dicot (%)			37.40	38.49	33.62
Total (%)			100.00	100.00	100.00

n- number of months

Table S4. Diet composition (percentage of occurrence) of swamp deer (*Rucervus duvaucelii*) during 2013-15 in Kaziranga National Park, Assam.

**Swamp Deer**					
**Plant species**	**Monocot/ Dicot**	**Growth form**	**Percentage of occurrence (%)**		
			**Overall (n=7)**	**Season**	
				**Dry (n=5)**	**Wet (n=2)**
Ziziphus jujuba Mill.	Dicot	Tree	5.10	5.15	4.94
Acmella uliginosa (Sw.) Cass.	Dicot	Herb	2.33	2.45	1.96
Lippia alba (Mill.) N.E.Br. ex Britton & P.Wilson	Dicot	Shrub	2.18	2.38	1.59
Solanum americanum Mill.	Dicot	Herb	1.87	1.81	2.07
Oxalis corniculata L.	Dicot	Herb	1.62	1.47	2.07
Polygonum plebeium R.Br.	Dicot	Herb	1.61	1.50	1.91
Persicaria hydropiper (L.) Delarbre	Dicot	Herb	1.53	1.43	1.80
Melilotus albus Medik.	Dicot	Herb	1.47	1.32	1.91
Chenopodium album L.	Dicot	Herb	1.43	1.25	1.96
Amaranthus viridis L.	Dicot	Herb	1.32	1.56	0.64
Mallotus nudiflorus (L.) Kulju & Welzen	Dicot	Tree	1.24	1.22	1.33
Duchesnea indica (Andrews) Focke	Dicot	Herb	1.00	1.04	0.90
Ranunculus sceleratus L.	Dicot	Herb	0.76	0.89	0.37
Leucas aspera (Willd.) Link	Dicot	Herb	0.75	0.80	0.58
Ludwigia adscendens (L.) H.Hara	Dicot	Herb	0.63	0.50	1.01
Acacia sp.	Dicot	Tree	0.63	0.66	0.53
Flemingia lineata (L.) Aiton	Dicot	Shrub	0.51	0.54	0.42
Oenanthe javanica (Blume) DC.	Dicot	Herb	0.48	0.34	0.90
Grangea maderaspatana (L.) Poir.	Dicot	Herb	0.43	0.38	0.58
Laphangium luteoalbum (L.) Tzvelev	Dicot	Herb	0.32	0.39	0.11
Pouzolzia zeylanica (L.) Benn. & R. Br.	Dicot	Herb	0.32	0.23	0.58
Nelsonia canescens (Lam.) Spreng.	Dicot	Herb	0.31	0.36	0.16
Cotula hemispherica (Roxb.) Raizada	Dicot	Herb	0.27	0.23	0.37
Solanum viarum Dunal	Dicot	Shrub	0.24	0.27	0.16
Merremia sp.	Dicot	Climber	0.21	0.21	0.21
Dalbergia sissoo DC.	Dicot	Tree	0.21	0.27	0.05
Litsea salicifolia (Roxburgh ex Nees) Hook. f.	Dicot	Shrub	0.17	0.16	0.21
Tetrastigma dubium (Lawson) Planch.	Dicot	Climber	0.12	0.16	0.00
Rungia pectinata (L.) Nees	Dicot	Herb	0.09	0.09	0.11
Rorippa indica (L.) Hiern	Dicot	Herb	0.08	0.05	0.16
Crotalaria albida Roth	Dicot	Shrub	0.07	0.04	0.16
Dillenia indica L.	Dicot	Tree	0.05	0.07	0.00
Hypericum sp.	Dicot	Shrub	0.04	0.05	0.00
Cannabis sativa L.	Dicot	Herb	0.03	0.04	0.00
Urena lobata L.	Dicot	Shrub	0.03	0.04	0.00
Amaranthus spinosus L.	Dicot	Herb	0.01	0.02	0.00
Xanthium strumarium L.	Dicot	Herb	0.01	0.00	0.05
Mikania micrantha Kunth	Dicot	Climber	0.01	0.00	0.05
Artemisia sp.	Dicot	Herb	0.01	0.02	0.00
Hemarthria compressa (L.f.) R.Br.	Monocot	Grass	9.95	10.68	7.80
Saccharum spp.	Monocot	Grass	9.62	10.12	8.12
Echinochloa crus-galli (L.) P.Beauv.	Monocot	Grass	6.66	6.42	7.38
Carex vesicaria L.	Monocot	Sedge	6.58	6.37	7.22
Imperata cylindrica (L.) Raeusch.	Monocot	Grass	5.23	4.83	6.42

Swamp Deer					
Plant species	Monocot/ Dicot	Growth form	Percentage of occurrence (%)		
			Overall (n=7)	Season	
				Dry (n=5)	Wet (n=2)
Cynodon dactylon (L.) Pers.	Monocot	Grass	4.48	4.88	3.29
Kyllinga brevifolia Rottb.	Monocot	Sedge	4.24	4.36	3.87
Fimbristylis aestivalis Vahl	Monocot	Sedge	2.92	3.00	2.65
Setaria pumila (Poir.) Roem. & Schult.	Monocot	Grass	1.83	1.79	1.96
Eleusine indica (L.) Gaertn.	Monocot	Grass	1.71	1.73	1.65
Eleocharis acutangula (Roxb.) Schult.	Monocot	Sedge	1.32	0.91	2.55
Alpinia nigra (Gaertn.) Burtt	Monocot	Herb	1.18	1.04	1.59
Paspalum conjugatum P.J.Bergius	Monocot	Grass	1.04	0.93	1.38
Chrysopogon aciculatus (Retz.) Trin.	Monocot	Grass	0.74	0.75	0.69
*Oplismenus*sp.	Monocot	Grass	0.43	0.29	0.85
Cyrtococcum sp.	Monocot	Grass	0.39	0.34	0.53
Fimbristylis dichotoma (L.) Vahl	Monocot	Sedge	0.25	0.11	0.69
Setaria sp.	Monocot	Grass	0.08	0.11	0.00
Panicum sp.	Monocot	Grass	0.04	0.05	0.00
Cyperus squarrosus L.	Monocot	Sedge	0.01	0.02	0.00
Unidentified - 14	Dicot	Unidentified	0.49	0.34	0.96
Unidentified - 4	Dicot	Unidentified	0.48	0.55	0.27
Unidentified - 11	Dicot	Unidentified	0.39	0.47	0.16
Unidentified - 13	Dicot	Unidentified	0.20	0.23	0.11
Unidentified - 23	Dicot	Unidentified	0.17	0.18	0.16
Unidentified - 8	Dicot	Unidentified	0.13	0.14	0.11
Unidentified - 16	Dicot	Unidentified	0.08	0.07	0.11
Unidentified - 18	Dicot	Unidentified	0.07	0.09	0.00
Unidentified - 29	Dicot	Unidentified	0.05	0.07	0.00
Unidentified - 27	Dicot	Unidentified	0.01	0.02	0.00
Unidentified - 31	Dicot	Unidentified	0.01	0.02	0.00
Unidentified - 32	Dicot	Unidentified	0.01	0.02	0.00
Unidentified - 3	Monocot	Unidentified	1.44	1.41	1.54
Unidentified - 7	Monocot	Unidentified	1.31	1.20	1.65
Unidentified - 2	Monocot	Unidentified	1.24	1.41	0.74
Unidentified - 1	Monocot	Unidentified	0.78	0.82	0.64
Unidentified - 25	Monocot	Unidentified	0.58	0.41	1.06
Unidentified - 5	Monocot	Unidentified	0.51	0.61	0.21
Unidentified - 9	Monocot	Unidentified	0.51	0.36	0.96
Unidentified - 15	Monocot	Unidentified	0.49	0.52	0.42
Unidentified - 12	Monocot	Unidentified	0.47	0.55	0.21
Unidentified - 26	Monocot	Unidentified	0.47	0.45	0.53
Unidentified - 10	Monocot	Unidentified	0.37	0.38	0.37
Unidentified - 21	Monocot	Unidentified	0.31	0.39	0.05
Unidentified - 20	Monocot	Unidentified	0.24	0.21	0.32
Unidentified - 24	Monocot	Unidentified	0.24	0.27	0.16
Unidentified - 6	Monocot	Unidentified	0.19	0.18	0.21
Unidentified - 17	Monocot	Unidentified	0.19	0.25	0.00
Unidentified - 28	Monocot	Unidentified	0.19	0.16	0.27
Unidentified - 19	Monocot	Unidentified	0.12	0.09	0.21
Unidentified - 30	Monocot	Unidentified	0.01	0.00	0.05
Identified Monocot (%)			58.72	58.74	58.65

Swamp Deer					
Plant species	Monocot/ Dicot	Growth form	Percentage of occurrence (%)		
			Overall (n=7)	Season	
				Dry (n=5)	Wet (n=2)
Unidentified Monocot (%)			9.66	9.68	9.61
Total Monocot (%)			68.37	68.41	68.26
Identified Dicot (%)			29.51	29.39	29.88
Unidentified Dicot (%)			2.11	2.20	1.86
Total Dicot (%)			31.63	31.59	31.74
Total (%)			100.00	100.00	100.00

n- number of months

Table S5. Diet composition (percentage of occurrence) of hog deer (*Axis porcinus*) during 2013-15 in Kaziranga National Park, Assam.

Hog Deer					
Plant species	Monocot/ Dicot	Growth form	Percentage of occurrence (%)		
			Overall (n=7)	Season	
				Dry (n=5)	Wet (n=2)
Ziziphus jujuba Mill.	Dicot	Tree	6.22	6.69	4.55
Amaranthus viridis L.	Dicot	Herb	2.32	2.62	1.25
Duchesnea indica (Andrews) Focke	Dicot	Herb	2.16	2.35	1.49
Solanum americanum Mill.	Dicot	Herb	2.12	2.12	2.12
Lippia alba (Mill.) N.E.Br. ex Britton & P.Wilson	Dicot	Shrub	2.11	2.14	1.99
Acmella uliginosa (Sw.) Cass.	Dicot	Herb	2.02	2.12	1.68
Oxalis corniculata L.	Dicot	Herb	1.75	1.88	1.31
Tetrastigma dubium (Lawson) Planch.	Dicot	Climber	1.55	1.44	1.93
Mallotus nudiflorus (L.) Kulju & Welzen	Dicot	Tree	1.25	1.23	1.31
Acacia sp.	Dicot	Tree	1.20	1.32	0.75
Polygonum plebeium R.Br.	Dicot	Herb	1.18	1.20	1.12
Flemingia lineata (L.) Aiton	Dicot	Shrub	1.06	1.04	1.12
Melilotus albus Medik.	Dicot	Herb	0.98	1.11	0.50
Persicaria hydropiper (L.) Delarbre	Dicot	Herb	0.88	0.96	0.62
Hypericum sp.	Dicot	Shrub	0.73	0.43	1.81
Dillenia indica L.	Dicot	Tree	0.71	0.75	0.56
Nelsonia canescens (Lam.) Spreng.	Dicot	Herb	0.69	0.71	0.62
Leucas aspera (Willd.) Link	Dicot	Herb	0.67	0.63	0.81
Chenopodium album L.	Dicot	Herb	0.61	0.75	0.12
Grangea maderaspatana (L.) Poir.	Dicot	Herb	0.53	0.31	1.31
Oenanthe javanica (Blume) DC.	Dicot	Herb	0.45	0.45	0.44
Ageratum conyzoides (L.) L.	Dicot	Herb	0.43	0.52	0.12
Laphangium luteoalbum (L.) Tzvelev	Dicot	Herb	0.38	0.37	0.44
Dalbergia sissoo DC.	Dicot	Tree	0.34	0.31	0.44
Pouzolzia zeylanica (L.) Benn. & R. Br.	Dicot	Herb	0.31	0.38	0.06
Crotalaria albida Roth	Dicot	Shrub	0.30	0.37	0.06
Solanum viarum Dunal	Dicot	Shrub	0.29	0.30	0.25
Urena lobata L.	Dicot	Shrub	0.24	0.30	0.06
Cannabis sativa L.	Dicot	Herb	0.24	0.30	0.06
Ranunculus sceleratus L.	Dicot	Herb	0.18	0.19	0.12
Rungia pectinata (L.) Nees	Dicot	Herb	0.15	0.16	0.12
Litsea salicifolia (Roxburgh ex Nees) Hook. f.	Dicot	Shrub	0.15	0.12	0.25
Amaranthus spinosus L.	Dicot	Herb	0.14	0.17	0.00
Cajanus scarabaeoides (L.) Thouars	Dicot	Climber	0.14	0.16	0.06
Mikania micrantha Kunth	Dicot	Climber	0.12	0.10	0.19
Merremia sp.	Dicot	Climber	0.11	0.07	0.25
Ludwigia adscendens (L.) H.Hara	Dicot	Herb	0.05	0.03	0.12
Cotula hemispherica (Roxb.) Raizada	Dicot	Herb	0.04	0.03	0.06
Youngia japonica (L.) DC.	Dicot	Herb	0.03	0.03	0.00
Merremia umbellata (L.) Hallier f.	Dicot	Climber	0.01	0.02	0.00
Hemarthria compressa (L.f.) R.Br.	Monocot	Grass	9.96	9.32	12.27
Saccharum spp.	Monocot	Grass	9.35	8.13	13.70
Echinochloa crus-galli (L.) P.Beauv.	Monocot	Grass	6.81	7.13	5.67

Hog Deer					
**Plant species**	**Monocot/ Dicot**	**Growth form**	**Percentage of occurrence (%)**		
			**Overall (n=7)**	**Season**	
				**Dry (n=5)**	**Wet (n=2)**
Carex vesicaria L.	Monocot	Sedge	5.88	6.05	5.29
Cynodon dactylon (L.) Pers.	Monocot	Grass	4.95	5.21	3.99
Kyllinga brevifolia Rottb.	Monocot	Sedge	3.04	3.13	2.74
Fimbristylis aestivalis Vahl	Monocot	Sedge	2.11	2.03	2.37
Setaria pumila (Poir.) Roem. & Schult.	Monocot	Grass	2.09	2.16	1.87
Imperata cylindrica (L.) Raeusch.	Monocot	Grass	1.86	1.72	2.37
Alpinia nigra (Gaertn.) Burtt	Monocot	Herb	1.82	1.79	1.93
Eleusine indica (L.) Gaertn.	Monocot	Grass	1.17	1.10	1.43
Paspalum conjugatum P.J.Bergius	Monocot	Grass	0.90	0.97	0.62
Eleocharis acutangula (Roxb.) Schult.	Monocot	Sedge	0.77	0.76	0.81
Chrysopogon aciculatus (Retz.) Trin.	Monocot	Grass	0.46	0.52	0.25
Panicum sp.	Monocot	Grass	0.31	0.33	0.25
Cyrtococcum sp.	Monocot	Grass	0.31	0.33	0.25
Setaria sp.	Monocot	Grass	0.24	0.21	0.37
*Oplismenus*sp.	Monocot	Grass	0.20	0.16	0.37
Fimbristylis dichotoma (L.) Vahl	Monocot	Sedge	0.12	0.16	0.00
Unidentified - 11	Dicot	Unidentified	0.39	0.43	0.25
Unidentified - 26	Dicot	Unidentified	0.38	0.31	0.62
Unidentified - 12	Dicot	Unidentified	0.37	0.30	0.62
Unidentified - 9	Dicot	Unidentified	0.31	0.38	0.06
Unidentified - 8	Dicot	Unidentified	0.30	0.31	0.25
Unidentified - 2	Dicot	Unidentified	0.11	0.14	0.00
Unidentified - 14	Dicot	Unidentified	0.11	0.12	0.06
Unidentified - 10	Dicot	Unidentified	0.10	0.10	0.06
Unidentified - 18	Dicot	Unidentified	0.07	0.03	0.19
Unidentified - 21	Dicot	Unidentified	0.07	0.07	0.06
Unidentified - 31	Dicot	Unidentified	0.03	0.03	0.00
Unidentified - 25	Dicot	Unidentified	0.01	0.02	0.00
Unidentified - 32	Dicot	Unidentified	0.01	0.00	0.06
Unidentified - 1	Monocot	Unidentified	1.59	1.60	1.56
Unidentified - 3	Monocot	Unidentified	1.59	1.62	1.49
Unidentified - 7	Monocot	Unidentified	1.59	1.48	1.99
Unidentified - 4	Monocot	Unidentified	1.52	1.62	1.18
Unidentified - 24	Monocot	Unidentified	0.46	0.43	0.56
Unidentified - 22	Monocot	Unidentified	0.45	0.37	0.75
Unidentified - 16	Monocot	Unidentified	0.43	0.42	0.50
Unidentified - 17	Monocot	Unidentified	0.43	0.43	0.44
Unidentified - 23	Monocot	Unidentified	0.42	0.43	0.37
Unidentified - 27	Monocot	Unidentified	0.39	0.23	1.00
Unidentified - 5	Monocot	Unidentified	0.33	0.35	0.25
Unidentified - 20	Monocot	Unidentified	0.29	0.30	0.25
Unidentified - 13	Monocot	Unidentified	0.24	0.26	0.19
Unidentified - 6	Monocot	Unidentified	0.23	0.30	0.00
Unidentified - 30	Monocot	Unidentified	0.20	0.17	0.31
Unidentified - 15	Monocot	Unidentified	0.19	0.23	0.06
Unidentified - 19	Monocot	Unidentified	0.05	0.07	0.00
Unidentified - 29	Monocot	Unidentified	0.05	0.05	0.06

Hog Deer					
**Plant species**	**Monocot/ Dicot**	**Growth form**	**Percentage of occurrence (%)**		
			**Overall (n=7)**	**Season**	
				**Dry (n=5)**	**Wet (n=2)**
Unidentified - 28	Monocot	Unidentified	0.04	0.00	0.19
Identified Monocot (%)			52.37	51.21	56.54
Unidentified Monocot (%)			10.52	10.34	11.15
Total Monocot (%)			62.89	61.55	67.68
Identified Dicot (%)			34.86	36.19	30.07
Unidentified Dicot (%)			2.26	2.26	2.24
Total Dicot (%)			37.11	38.45	32.32
Total (%)			100.00	100.00	100.00

n- number of months

Table S6. Diet composition (percentage of occurrence) of sambar (*Rusa unicolor*) during 2013-15 in Kaziranga National Park, Assam from 2013 to 2015.

Sambar					
**Plant species**	**Monocot / Dicot**	**Growth form**	**Percentage of occurrence (%)**		
			**Overall (n=7)**	**Season**	
				**Dry (n=5)**	**Wet (n=2)**
Ziziphus jujuba Mill.	Dicot	Tree	7.59	8.05	5.16
Acmella uliginosa (Sw.) Cass.	Dicot	Herb	2.69	2.82	1.96
Solanum americanum Mill.	Dicot	Herb	2.57	2.62	2.32
Dillenia indica L.	Dicot	Tree	2.33	2.50	1.42
Mallotus nudiflorus (L.) Kulju & Welzen	Dicot	Tree	2.09	2.20	1.51
Lippia alba (Mill.) N.E.Br. ex Britton & P.Wilson	Dicot	Shrub	2.05	2.25	0.98
Melilotus albus Medik.	Dicot	Herb	1.95	2.01	1.60
Chenopodium album L.	Dicot	Herb	1.93	2.15	0.80
Amaranthus viridis L.	Dicot	Herb	1.78	1.84	1.42
Ageratum conyzoides (L.) L.	Dicot	Herb	1.65	1.83	0.71
Tetrastigma dubium (Lawson) Planch.	Dicot	Climber	1.65	1.78	0.98
Acacia sp.	Dicot	Tree	1.48	1.44	1.69
Duchesnea indica (Andrews) Focke	Dicot	Herb	1.45	1.59	0.71
Nelsonia canescens (Lam.) Spreng.	Dicot	Herb	1.44	1.62	0.45
Hypericum sp.	Dicot	Shrub	1.32	1.35	1.16
Urena lobata L.	Dicot	Shrub	1.32	1.39	0.98
Merremia sp.	Dicot	Climber	1.25	1.32	0.89
Leucas aspera (Willd.) Link	Dicot	Herb	1.21	1.22	1.16
Persicaria hydropiper (L.) Delarbre	Dicot	Herb	1.08	1.10	0.98
Polygonum plebeium R.Br.	Dicot	Herb	0.74	0.79	0.45
Oenanthe javanica (Blume) DC.	Dicot	Herb	0.71	0.79	0.27
Crotalaria albida Roth	Dicot	Shrub	0.70	0.78	0.27
Pouzolzia zeylanica (L.) Benn. & R. Br.	Dicot	Herb	0.53	0.61	0.09
Laphangium luteoalbum (L.) Tzvelev	Dicot	Herb	0.50	0.49	0.53
Amaranthus spinosus L.	Dicot	Herb	0.48	0.46	0.62
Dalbergia sissoo DC.	Dicot	Tree	0.47	0.52	0.18
Ludwigia adscendens (L.) H.Hara	Dicot	Herb	0.43	0.44	0.36
Oxalis corniculata L.	Dicot	Herb	0.43	0.44	0.36
Cannabis sativa L.	Dicot	Herb	0.36	0.36	0.36
Flemingia lineata (L.) Aiton	Dicot	Shrub	0.33	0.34	0.27
Grangea maderaspatana (L.) Poir.	Dicot	Herb	0.31	0.37	0.00
Litsea salicifolia (Roxburgh ex Nees) Hook. f.	Dicot	Shrub	0.23	0.25	0.09
Ranunculus sceleratus L.	Dicot	Herb	0.23	0.25	0.09
Glochidion multiloculare (Rottler ex Willd.) Voigt	Dicot	Shrub	0.18	0.15	0.36
Flueggea virosa (Roxb. ex Willd.) Royl	Dicot	Shrub	0.18	0.15	0.36
Solanum viarum Dunal	Dicot	Shrub	0.18	0.20	0.09
Mikania micrantha Kunth	Dicot	Climber	0.17	0.19	0.09
Cotula hemispherica (Roxb.) Raizada	Dicot	Herb	0.09	0.08	0.09
Youngia japonica (L.) DC.	Dicot	Herb	0.06	0.07	0.00
Rungia pectinata (L.) Nees	Dicot	Herb	0.04	0.03	0.09
Rorippa indica (L.) Hiern	Dicot	Herb	0.04	0.05	0.00
Cajanus scarabaeoides (L.) Thouars	Dicot	Climber	0.03	0.03	0.00
Cleome spinosa Jacq.	Dicot	Herb	0.01	0.02	0.00
Rumex dentatus L.	Dicot	Herb	0.01	0.02	0.00

Sambar					
Plant species	Monocot / Dicot	Growth form	Percentage of occurrence (%)		
			Overall (n=7)	Season	
				Dry (n=5)	Wet (n=2)
Stachytarpheta indica (L.) Vahl	Dicot	Shrub	0.01	0.02	0.00
Saccharum spp.	Monocot	Grass	13.40	12.44	18.43
Echinochloa crus-galli (L.) P.Beauv.	Monocot	Grass	5.23	5.09	5.97
Hemarthria compressa (L.f.) R.Br.	Monocot	Grass	4.97	4.82	5.79
Carex vesicaria L.	Monocot	Sedge	4.66	4.14	7.39
Imperata cylindrica (L.) Raeusch.	Monocot	Grass	2.20	1.99	3.29
Kyllinga brevifolia Rottb.	Monocot	Sedge	2.13	2.01	2.76
Cynodon dactylon (L.) Pers.	Monocot	Grass	1.95	1.99	1.69
Fimbristylis aestivalis Vahl	Monocot	Sedge	1.86	1.96	1.34
Alpinia nigra (Gaertn.) Burtt	Monocot	Herb	1.75	1.72	1.87
Chrysopogon aciculatus (Retz.) Trin.	Monocot	Grass	1.17	1.10	1.51
Setaria pumila (Poir.) Roem. & Schult.	Monocot	Grass	1.17	1.12	1.42
Eleocharis acutangula (Roxb.) Schult.	Monocot	Sedge	1.05	0.81	2.32
Eleusine indica (L.) Gaertn.	Monocot	Grass	0.54	0.46	0.98
Paspalum conjugatum P.J.Bergius	Monocot	Grass	0.38	0.41	0.27
Cyrtococcum sp.	Monocot	Grass	0.30	0.19	0.89
Fimbristylis dichotoma (L.) Vahl	Monocot	Sedge	0.11	0.03	0.53
Setaria sp.	Monocot	Grass	0.04	0.05	0.00
*Oplismenus*sp.	Monocot	Grass	0.03	0.03	0.00
Unidentified - 2	Dicot	Unidentified	0.82	0.86	0.62
Unidentified - 8	Dicot	Unidentified	0.43	0.46	0.27
Unidentified - 14	Dicot	Unidentified	0.38	0.37	0.45
Unidentified - 15	Dicot	Unidentified	0.16	0.17	0.09
Unidentified - 3	Dicot	Unidentified	0.10	0.10	0.09
Unidentified - 22	Dicot	Unidentified	0.09	0.08	0.09
Unidentified - 26	Dicot	Unidentified	0.09	0.10	0.00
Unidentified - 5	Dicot	Unidentified	0.07	0.08	0.00
Unidentified - 23	Dicot	Unidentified	0.07	0.08	0.00
Unidentified - 28	Dicot	Unidentified	0.07	0.08	0.00
Unidentified - 29	Dicot	Unidentified	0.01	0.02	0.00
Unidentified - 4	Monocot	Unidentified	1.56	1.59	1.42
Unidentified - 10	Monocot	Unidentified	1.15	1.27	0.53
Unidentified - 1	Monocot	Unidentified	1.09	1.05	1.34
Unidentified - 11	Monocot	Unidentified	0.90	0.59	2.49
Unidentified - 13	Monocot	Unidentified	0.77	0.69	1.16
Unidentified - 20	Monocot	Unidentified	0.68	0.66	0.80
Unidentified - 6	Monocot	Unidentified	0.45	0.41	0.71
Unidentified - 7	Monocot	Unidentified	0.44	0.46	0.36
Unidentified - 9	Monocot	Unidentified	0.33	0.39	0.00
Unidentified - 25	Monocot	Unidentified	0.26	0.25	0.27
Unidentified - 19	Monocot	Unidentified	0.17	0.17	0.18
Unidentified - 12	Monocot	Unidentified	0.16	0.10	0.45
Unidentified - 17	Monocot	Unidentified	0.13	0.15	0.00
Unidentified - 18	Monocot	Unidentified	0.13	0.14	0.09
Unidentified - 16	Monocot	Unidentified	0.11	0.08	0.27
Unidentified - 27	Monocot	Unidentified	0.11	0.14	0.00
Unidentified - 21	Monocot	Unidentified	0.04	0.05	0.00

Sambar					
**Plant species**	**Monocot / Dicot**	**Growth form**	**Percentage of occurrence (%)**		
			**Overall (n=7)**	**Season**	
				**Dry (n=5)**	**Wet (n=2)**
Unidentified - 24	Monocot	Unidentified	0.04	0.05	0.00
Identified Monocot (%)			42.94	40.37	56.46
Unidentified Monocot (%)			8.53	8.23	10.06
Total Monocot (%)			51.46	48.61	66.52
Identified Dicot (%)			46.25	48.98	31.88
Unidentified Dicot (%)			2.29	2.42	1.60
Total Dicot (%)			48.54	51.39	33.48
Total (%)			100.00	100.00	100.00

n- number of months

Table S7. Overall (throughout the year) nutrient content (percentage) of major forage plants consumed by mega and meso-herbivores during 2013-15 in Kaziranga National Park, Assam.

Plant species	Monocot/ Dicot	AC (%)	CP (%)	ADF (%)	ADL (%)	NDF (%)	Ca (%)	Mg (%)	Na (%)	K (%)	P (%)
Alpinia nigra (Gaertn.) Burtt	Monocot	10.63	7.57	55.76	17.91	71.09	0.83	0.21	0.04	2.58	0.09
Calamus tenuis Roxb.	Monocot	8.02	11.42	53.90	19.15	75.03	0.64	0.12	0.03	1.08	0.06
Carex vesicaria L.	Monocot	11.09	8.92	50.27	12.11	71.34	1.11	0.22	0.04	1.59	0.11
Cynodon dactylon (L.) Pers.	Monocot	11.88	7.47	46.70	9.59	73.41	0.86	0.29	0.04	1.50	0.09
Echinochloa crus-galli (L.) P.Beauv.	Monocot	16.17	12.16	40.66	11.22	60.62	1.22	0.44	0.06	1.65	0.13
Hemarthria compressa (L.f.) R.Br.	Monocot	11.20	7.19	50.40	11.08	67.01	0.86	0.57	0.06	1.92	0.20
Imperata cylindrica (L.) Raeusch.	Monocot	8.14	6.63	57.54	11.85	75.34	1.04	0.18	0.03	0.90	0.07
Saccharum spp.	Monocot	8.03	6.02	60.52	11.70	75.21	0.71	0.22	0.03	0.89	0.06
**Mean monocots**		**10.65**	**8.42**	**51.97**	**13.08**	**71.13**	**0.91**	**0.28**	**0.04**	**1.51**	**0.10**
**Standard deviation**		**2.74**	**2.25**	**6.36**	**3.47**	**5.10**	**0.20**	**0.15**	**0.01**	**0.57**	**0.05**
Ageratum conyzoides (L.) L.	Dicot	13.84	12.88	49.87	16.76	59.43	1.73	0.48	0.08	2.37	0.22
Amaranthus viridis L.	Dicot	19.31	17.88	35.33	16.80	49.81	2.00	1.08	0.09	4.38	0.41
Dillenia indica L.	Dicot	12.08	6.94	55.85	13.52	66.78	1.43	0.26	0.05	1.30	0.07
Lippia alba (Mill.) N.E.Br. ex Britton & P.Wilson	Dicot	10.75	9.49	53.16	14.40	63.48	1.93	0.29	0.07	1.36	0.19
Litsea salicifolia (Roxburgh ex Nees) Hook. f.	Dicot	5.49	9.05	66.65	13.66	72.42	1.24	0.21	0.04	0.86	0.11
Mallotus nudiflorus (L.) Kulju & Welzen	Dicot	8.37	13.17	46.90	14.24	59.88	1.48	0.24	0.05	1.58	0.24
Oxalis corniculata L.	Dicot	13.68	15.04	46.12	17.11	55.95	1.30	0.45	0.08	1.64	0.22
Solanum americanum Mill.	Dicot	17.26	14.43	48.59	13.44	55.85	1.37	0.36	0.08	4.88	0.27
Ziziphus jujuba Mill.	Dicot	6.27	9.73	62.16	13.97	68.84	1.47	0.25	0.06	0.94	0.10
**Mean dicots**		**11.89**	**12.07**	**51.62**	**14.88**	**61.38**	**1.55**	**0.40**	**0.07**	**2.15**	**0.20**
**Standard deviation**		**4.71**	**3.49**	**9.27**	**1.54**	**7.17**	**0.27**	**0.27**	**0.02**	**1.48**	**0.11**
**Mean (Monocots & Dicots)**		**11.31**	**10.35**	**51.79**	**14.03**	**65.97**	**1.25**	**0.34**	**0.06**	**1.85**	**0.16**
**Standard deviation**		**3.84**	**3.44**	**7.79**	**2.70**	**7.89**	**0.40**	**0.23**	**0.02**	**1.16**	**0.10**

AC- ash content, CP- crude protein; ADF- acid detergent fibre; ADL- acid detergent lignin, NDF- neutral detergent fibre, Ca- calcium, P- phosphorous, Mg- magnesium, K- potassium and Na- sodium.

Table S8. Dry season nutrient content (percentage) of major forage plants consumed by mega and meso-herbivores during 2013-15 in Kaziranga National Park, Assam.

Plant species	Monocot/ Dicot	AC (%)	CP (%)	ADF (%)	ADL (%)	NDF (%)	Ca (%)	Mg (%)	Na (%)	K (%)	P (%)
Alpinia nigra (Gaertn.) Burtt	Monocot	10.68	6.96	57.59	18.96	72.09	0.81	0.21	0.04	2.46	0.08
Calamus tenuis Roxb.	Monocot	8.32	10.87	53.21	19.77	75.76	0.66	0.10	0.03	0.98	0.06
Carex vesicaria L.	Monocot	12.72	8.25	51.69	13.16	69.89	1.07	0.20	0.03	1.38	0.08
Cynodon dactylon (L.) Pers.	Monocot	13.51	6.70	48.24	10.52	72.66	0.86	0.25	0.04	0.99	0.06
Echinochloa crus-galli (L.) P.Beauv.	Monocot	17.31	10.44	41.93	12.79	61.88	0.88	0.33	0.04	1.19	0.10
Hemarthria compressa (L.f.) R.Br.	Monocot	12.65	6.35	52.40	12.50	68.97	0.72	0.47	0.05	1.39	0.17
Imperata cylindrica (L.) Raeusch.	Monocot	8.77	5.59	57.41	13.74	75.94	1.00	0.19	0.03	0.71	0.04
Saccharum spp.	Monocot	9.35	4.75	61.46	13.13	76.15	0.63	0.20	0.03	0.80	0.05
**Mean monocots**		**11.66**	**7.49**	**52.99**	**14.32**	**71.67**	**0.83**	**0.24**	**0.04**	**1.24**	**0.08**
**Standard deviation**		**3.00**	**2.20**	**6.09**	**3.26**	**4.82**	**0.16**	**0.11**	**0.01**	**0.55**	**0.04**
Ageratum conyzoides (L.) L.	Dicot	14.25	13.50	49.44	17.41	57.69	1.56	0.45	0.08	2.01	0.18
Amaranthus viridis L.	Dicot	18.84	17.40	35.75	17.04	49.66	1.87	0.95	0.08	4.17	0.37
Dillenia indica L.	Dicot	12.45	6.80	54.53	14.10	66.61	1.58	0.26	0.05	1.23	0.06
Lippia alba (Mill.) N.E.Br. ex Britton & P.Wilson	Dicot	10.39	9.34	54.10	14.80	62.32	2.04	0.28	0.06	1.27	0.18
Litsea salicifolia (Roxburgh ex Nees) Hook. f.	Dicot	5.20	9.02	66.44	13.91	72.10	1.34	0.20	0.04	0.77	0.10
Mallotus nudiflorus (L.) Kulju & Welzen	Dicot	8.43	12.60	45.42	14.14	59.45	1.50	0.23	0.05	1.53	0.22
Oxalis corniculata L.	Dicot	13.77	15.49	44.78	17.44	56.05	1.30	0.45	0.08	1.62	0.21
Solanum americanum Mill.	Dicot	17.35	16.77	46.67	14.19	53.25	1.44	0.38	0.08	4.98	0.26
Ziziphus jujuba Mill.	Dicot	6.42	9.43	61.69	14.94	68.09	1.54	0.27	0.06	0.89	0.09
**Mean dicots**		**11.90**	**12.26**	**50.98**	**15.33**	**60.58**	**1.58**	**0.38**	**0.06**	**2.05**	**0.19**
**Standard deviation**		**4.70**	**3.80**	**9.34**	**1.52**	**7.35**	**0.24**	**0.23**	**0.02**	**1.49**	**0.10**
**Mean (Monocots & Dicots)**		**11.79**	**10.01**	**51.93**	**14.86**	**65.80**	**1.22**	**0.32**	**0.05**	**1.67**	**0.14**
**Standard deviation**		**3.87**	**3.92**	**7.81**	**2.46**	**8.35**	**0.43**	**0.19**	**0.02**	**1.19**	**0.09**

AC- ash content, CP- crude protein; ADF- acid detergent fibre; ADL- acid detergent lignin, NDF- neutral detergent fibre, Ca- calcium, P- phosphorous, Mg- magnesium, K- potassium and Na- sodium.

Table S9. Wet season nutrient content (percentage) of major forage plants consumed by mega and meso-herbivores during 2013-15 in Kaziranga National Park, Assam.

**Plant species**	**Monocot/ Dicot**	**AC (%)**	**CP (%)**	**ADF (%)**	**ADL (%)**	**NDF (%)**	**Ca (%)**	**Mg (%)**	**Na (%)**	**K (%)**	**P (%)**
Alpinia nigra (Gaertn.) Burtt	Monocot	10.50	9.11	51.18	15.30	68.59	0.89	0.22	0.04	2.90	0.14
Calamus tenuis Roxb.	Monocot	7.28	12.80	55.61	17.61	73.21	0.61	0.17	0.04	1.34	0.07
Carex vesicaria L.	Monocot	7.02	10.59	46.71	9.48	74.97	1.21	0.26	0.04	2.13	0.17
Cynodon dactylon (L.) Pers.	Monocot	7.81	9.38	42.84	7.28	75.27	0.89	0.39	0.05	2.79	0.16
Echinochloa crus-galli (L.) P.Beauv.	Monocot	13.32	16.47	37.50	7.30	57.49	2.07	0.72	0.09	2.82	0.20
Hemarthria compressa (L.f.) R.Br.	Monocot	7.57	9.30	45.40	7.52	62.11	1.20	0.83	0.08	3.24	0.28
Imperata cylindrica (L.) Raeusch.	Monocot	6.56	9.24	57.87	7.13	73.82	1.14	0.17	0.04	1.36	0.14
Saccharum spp.	Monocot	4.74	9.19	58.16	8.13	72.87	0.90	0.26	0.05	1.11	0.09
**Mean monocots**		**8.10**	**10.76**	**49.41**	**9.97**	**69.79**	**1.11**	**0.38**	**0.05**	**2.21**	**0.16**
**Standard deviation**		**2.64**	**2.63**	**7.54**	**4.12**	**6.61**	**0.44**	**0.26**	**0.02**	**0.84**	**0.07**
Ageratum conyzoides (L.) L.	Dicot	12.81	11.33	50.94	15.12	63.79	2.16	0.57	0.10	3.27	0.32
Amaranthus viridis L.	Dicot	20.49	19.09	34.28	16.19	50.17	2.30	1.40	0.10	4.92	0.51
Dillenia indica L.	Dicot	11.17	7.26	59.15	12.06	67.21	1.06	0.24	0.05	1.49	0.07
Lippia alba (Mill.) N.E.Br. ex Britton & P.Wilson	Dicot	11.62	9.87	50.82	13.39	66.38	1.65	0.31	0.08	1.58	0.22
Litsea salicifolia (Roxburgh ex Nees) Hook. f.	Dicot	6.20	9.11	67.16	13.05	73.23	1.01	0.23	0.05	1.07	0.16
Mallotus nudiflorus (L.) Kulju & Welzen	Dicot	8.21	14.61	50.60	14.50	60.96	1.43	0.27	0.06	1.70	0.28
Oxalis corniculata L.	Dicot	13.47	13.91	49.47	16.29	55.69	1.32	0.47	0.08	1.70	0.25
Solanum americanum Mill.	Dicot	17.02	8.58	53.39	11.58	62.36	1.19	0.30	0.09	4.64	0.29
Ziziphus jujuba Mill.	Dicot	5.88	10.46	63.33	11.52	70.72	1.28	0.21	0.07	1.05	0.12
**Mean dicots**		**11.87**	**11.58**	**53.24**	**13.74**	**63.39**	**1.49**	**0.44**	**0.08**	**2.38**	**0.25**
**Standard deviation**		**4.82**	**3.69**	**9.50**	**1.87**	**7.21**	**0.46**	**0.38**	**0.02**	**1.51**	**0.13**
**Mean (Monocots & Dicots)**		**10.10**	**11.19**	**51.44**	**11.97**	**66.40**	**1.31**	**0.41**	**0.07**	**2.30**	**0.20**
**Standard deviation**		**4.30**	**3.16**	**8.60**	**3.60**	**7.48**	**0.48**	**0.32**	**0.02**	**1.21**	**0.11**

AC- ash content, CP- crude protein; ADF- acid detergent fibre; ADL- acid detergent lignin, NDF- neutral detergent fibre, Ca- calcium, P- phosphorous, Mg- magnesium, K- potassium and Na- sodium.

Table S10. Seasonal differences in the nutrient content of major forage consumed by mega and meso-herbivores during 2013-15 in Kaziranga National Park, Assam.

Forage	Season	Parameter	Mann-Whitney (U)	p
Monocot	Between dry and Wet Season	AC	9	**0.015**
		CP	11	**0.028**
		ADF	24	0.442
		ADL	12	**0.038**
		NDF	27	0.645
		Ca	14	0.065
		Mg	22	0.328
		Na	9	**0.015**
		K	11	**0.028**
		P	9	**0.015**
Dicot	Between dry and Wet Season	AC	38	0.863
		CP	37	0.796
		ADF	32	0.489
		ADL	22	0.113
		NDF	31	0.436
		Ca	28	0.297
		Mg	38	0.863
		Na	26	0.222
		K	33	0.546
		P	27	0.258
Monocot and Dicot	Throughout the year	AC	109	0.237
		CP	80	**0.027**
		ADF	138	0.851
		ADL	86	**0.046**
		NDF	47	**0.001**
		Ca	26	**0.000**
		Mg	91	0.070
		Na	42	**0.000**
		K	115	0.330
		P	61	**0.003**
Monocot and Dicot	Dry Season	AC	34	0.847
		CP	10	**0.012**
		ADF	31	0.630
		ADL	18	0.083
		NDF	7	**0.005**
		Ca	0	**0.001**
		Mg	18	0.083
		Na	4	**0.002**
		K	22	0.178
		P	8	**0.007**
Monocot and Dicot	Wet Season	AC	19	0.102
		CP	33	0.773
		ADF	27	0.386
		ADL	16	0.054
		NDF	17	0.068
		Ca	15	**0.043**

Forage	Season	Parameter	Mann-Whitney (U)	p
		Mg	29	0.501
		Na	13	**0.027**
		K	36	1.000
		P	19	0.102
Monocot and Dicot	Between dry and Wet Season	AC	101	0.140
		CP	110	0.245
		ADF	143	0.973
		ADL	84	**0.038**
		NDF	139	0.865
		Ca	132	0.683
		Mg	120	0.413
		Na	84	**0.038**
		K	83	**0.034**
		P	86	**0.045**

AC- ash content, CP- crude protein; ADF- acid detergent fibre; ADL- acid detergent lignin, NDF- neutral detergent fibre, Ca- calcium, P- phosphorous, Mg- magnesium, K- potassium and Na- sodium.

Table S11. Model selection of major forage consumed by mega and meso-herbivores during 2013-15 in Kaziranga National Park, Assam.

Study Species	Season	Model	Ak. wt	AICc	delta AICc	df	Log L
Greater One-horned Rhino	Overall	CP+ADL	0.82	2.1	0	4	4.62
		CP+ADL+Ca	0.16	5.3	3.25	5	5.06
		CP+ADL+Ca+P	0.02	10.2	8.07	6	5.12
	Dry	CP+ADL	0.78	2.1	0	4	4.62
		CP+ADL+Ca	0.19	4.9	2.81	5	5.27
		CP+ADL+Ca+Na	0.03	8.6	6.5	6	5.91
	Wet	ADL+AC	0.88	0.5	0	4	5.43
		ADL+AC+P	0.11	4.6	4.12	5	5.44
		ADL+AC+P+NDF	0.01	9	8.52	6	5.71
Asian elephant	Overall	NDF+Na	0.87	3.8	0	4	3.77
		NDF+Na+P	0.12	7.8	3.99	5	3.83
		NDF+Na+P+Ca	0.01	12.3	8.48	6	4.06
	Dry	NDF+Na	0.88	5.4	0	4	2.95
		NDF+Na+P	0.11	9.5	4.11	5	2.95
		NDF+Na+P+Mg	0.01	14.4	8.97	6	3.00
	Wet	AC+ADL	0.88	1.2	0	4	5.05
		AC+ADL+P	0.11	5.3	4.1	5	5.06
		AC+ADL+P+K	0.01	10.2	8.99	6	5.09
Asiatic wild buffalo	Overall	ADL+Ca	0.86	1.5	0	4	4.93
		ADL+Ca+CP	0.12	5.4	3.95	5	5.02
		ADL+Ca+CP+NDF	0.02	9	7.58	6	5.68
	Dry	ADL+Ca	0.85	0.6	0	4	5.35
		ADL+Ca+CP	0.11	4.7	4.08	5	5.37
		ADL+Ca+CP+Na	0.04	6.9	6.29	6	6.74
	Wet	ADL+AC	0.61	1.7	0	4	4.83
		ADL+AC+ADF	0.36	2.7	1.04	5	6.37
		ADL+AC+ADF+P	0.03	7.7	5.98	6	6.37
Swamp Deer	Overall	ADL+CP	0.64	8.8	0	4	1.28
		ADL+CP+Ca	0.32	10.2	1.39	5	2.65
		ADL+CP+Ca+NDF	0.04	14.2	5.47	6	3.08
	Dry	ADL+Ca	0.85	5.4	0	4	2.97
		ADL+Ca+CP	0.11	9.4	4.03	5	3.02
		ADL+Ca+CP+NDF	0.04	11.6	6.21	6	4.40
	Wet	ADL+AC	0.77	5.7	0	4	2.79
		ADL+AC+P	0.15	9	3.21	5	3.25
		ADL+AC+P+NDF	0.07	10.5	4.78	6	4.94
Hog Deer	Overall	ADL+Ca	0.85	9	0	4	1.18
		ADL+Ca+CP	0.13	12.8	3.79	5	1.35
		ADL+Ca+CP+NDF	0.03	15.9	6.97	6	2.23
	Dry	ADL+Ca	0.80	9.9	0	4	0.72
		ADL+Ca+CP	0.11	14	4.06	5	0.75
		ADL+Ca+CP+Na	0.09	14.2	4.34	6	3.08
	Wet	ADL+AC	0.84	5.8	0	4	2.79

Study Species	Season	Model	Ak. wt	AICc	delta AICc	df	Log L
		ADL+AC+CP	0.14	9.3	3.54	5	3.08
		ADL+AC+CP+Ca	0.01	14.2	8.41	6	3.11
Sambar	Overall	ADL+CP	0.88	5.8	0	4	2.78
		ADL+CP+P	0.11	9.9	4.12	5	2.79
		ADL+CP+P+NDF	0.01	14.8	9.01	6	2.81
	Dry	ADL+CP	0.86	7	0	4	2.16
		ADL+CP+ADF	0.13	10.9	3.87	5	2.28
		ADL+CP+ADF+Ca	0.01	15.5	8.43	6	2.47
	Wet	ADL+AC	0.87	2.7	0	4	4.31
		ADL+AC+P	0.11	6.8	4.1	5	4.31
		ADL+AC+P+NDF	0.01	11	8.29	6	4.69

AC- ash content, CP- crude protein; ADF- acid detergent fibre; ADL- acid detergent lignin, NDF- neutral detergent fibre, Ca- calcium, P- phosphorous, Mg- magnesium, K- potassium and Na- sodium.

Table S12. List of forage plant samples collected and analyzed during 2013 to 2015 in Kaziranga National Park, Assam.

S. No.	Plant species	Family
1	*Nelsonia canescens* (Lam.) Spreng.	Acanthaceae
2	*Rungia pectinata* (L.) Nees	
3	*Amaranthus spinosus* L.	Amaranthaceae
4	*Chenopodium album* L.	
5	*Amaranthus viridis* L.	
6	*Oenanthe javanica* (Blume) DC.	Apiaceae
7	*Calamus tenuis* Roxb.	Arecaceae
8	*Heliotropium indicum* L.	Boraginaceae
9	*Rorippa indica* (L.) Hiern	Brassicaceae
10	*Capsella bursa-pastoris* (L.) Medik.	
11	*Cannabis sativa* L.	Cannabaceae
12	*Cleome spinosa* Jacq.	Cleomaceae
13	*Cotula hemispherica* (Roxb.) Raizada	Compositae
14	*Acmella uliginosa* (Sw.) Cass.	
15	*Ageratum conyzoides* (L.) L.	
16	*Laphangium luteoalbum* (L.) Tzvelev	
17	*Grangea maderaspatana* (L.) Poir.	
18	*Xanthium strumarium* L.	
19	*Mikania micrantha* Kunth	
20	*Youngia japonica* (L.) DC.	
21	*Crassocephalum crepidioides* (Benth.) S.Moore	
22	*Artemisia* sp.	
23	*Merremia* sp.	Convolvulaceae
24	*Merremia umbellata* (L.) Hallier f.	
25	*Carex vesicaria* L.	Cyperaceae
26	*Fimbristylis aestivalis* Vahl	
27	*Eleocharis acutangula* (Roxb.) Schult.	
28	*Fimbristylis dichotoma* (L.) Vahl	
29	*Kyllinga brevifolia* Rottb.	
30	*Cyperus squarrosus* L.	
31	*Dillenia indica* L.	Dilleniaceae
32	*Mallotus nudiflorus* (L.) Kulju & Welzen	Euphorbiaceae
33	*Hypericum* sp.	Hypericaceae
34	*Leucas aspera* (Willd.) Link	Lamiaceae
35	*Litsea salicifolia* (Roxburgh ex Nees) Hook. f.	Lauraceae
36	*Flemingia lineata* (L.) Aiton	Leguminosae
37	*Cajanus scarabaeoides* (L.) Thouars	
38	*Flemingia strobilifera* (L.) W.T.Aiton	
39	*Mimosa* spp.	

S. No.	Plant species	Family
40	*Vicia sativa* L.	
41	*Melilotus albus* Medik.	
42	*Crotalaria albida* Roth	
43	*Dalbergia sissoo* DC.	
44	*Acacia* sp.	
45	*Urena lobata* L.	Malvaceae
46	*Ludwigia adscendens* (L.) H.Hara	Onagraceae
47	*Oxalis corniculata* L.	Oxalidaceae
48	*Glochidion multiloculare* (Rottler ex Willd.) Voigt	Phyllanthaceae
49	*Flueggea virosa* (Roxb. ex Willd.) Royl	
50	*Cynodon dactylon* (L.) Pers.	Poaceae
51	*Echinochloa crus-galli* (L.) P.Beauv.	
52	*Panicum* sp.	
53	*Oplismenus*sp.	
54	*Cyrtococcum* sp.	
55	*Saccharum* spp.	
56	*Imperata cylindrica* (L.) Raeusch.	
57	*Setaria* sp.	
58	*Hemarthria compressa* (L.f.) R.Br.	
59	*Paspalum conjugatum* P.J.Bergius	
60	*Chrysopogon aciculatus* (Retz.) Trin.	
61	*Eleusine indica* (L.) Gaertn.	
62	*Setaria pumila* (Poir.) Roem. & Schult.	
63	*Polygonum plebeium* R.Br.	Polygonaceae
64	*Persicaria hydropiper* (L.) Delarbre	
65	*Rumex dentatus* L.	
66	*Ranunculus sceleratus* L.	Ranunculaceae
67	*Ziziphus jujuba* Mill.	Rhamnaceae
68	*Duchesnea indica* (Andrews) Focke	Rosaceae
69	*Solanum viarum* Dunal	Solanaceae
70	*Solanum americanum* Mill.	
71	*Pouzolzia zeylanica* (L.) Benn. & R. Br.	Urticaceae
72	*Lippia alba* (Mill.) N.E.Br. ex Britton & P.Wilson	Verbenaceae
73	*Stachytarpheta indica* (L.) Vahl	
74	*Tetrastigma dubium* (Lawson) Planch.	Vitaceae
75	*Alpinia nigra* (Gaertn.) Burtt	Zingiberaceae

Table S13. GPS coordinates of the faecal collection sites visited frequently for faecal sample collection of mega and meso-herbivores during 2013-15 in Kaziranga National Park, Assam.

S.No	Forest Range	Location	GPS coordinates					
			Degrees	Minutes	Seconds	Degrees	Minutes	Seconds
1	Agoratoli	Debeswari	26	43	39.5	93	28	7.8
2	Agoratoli	Dhuba	26	41	16.2	93	34	36.7
3	Agoratoli	Maklung	26	42	24.9	93	31	1.1
4	Agoratoli	Mohkhuti	26	40	45.2	93	33	49.2
5	Agoratoli	Mohpora	26	41	39.8	93	33	53.7
6	Agoratoli	Muamari	26	41	52.5	93	32	23.8
7	Agoratoli	Natunbeel	26	41	1.9	93	29	11.7
8	Agoratoli	Pahumari	26	41	25.8	93	31	21.5
9	Agoratoli	Rongamotia	26	41	57.1	93	30	56
10	Agoratoli	Sohola	26	40	29.3	93	32	54.5
11	Agoratoli	Tamulipathar	26	40	6.6	93	31	7.1
12	Agoratoli	Tinibeel	26	40	59.9	93	31	7.7
13	Agoratoli	Turturani	26	41	38.3	93	33	48.8
14	Kohora	Baghmari	26	39	18.6	93	21	17.2
15	Kohora	Baruntika	26	39	8.5	93	22	32.4
16	Kohora	Bokpora	26	37	52	93	22	46.6
17	Kohora	Dusuti	26	39	56.5	93	24	50.1
18	Kohora	Gobrai	26	40	29.6	93	19	15.3
19	Kohora	Hatikhuli Borbeel	26	39	25.8	93	22	15.8
20	Kohora	Holalpath	26	39	57.3	93	21	42.1
21	Kohora	Jamuguri	26	39	9.8	93	22	27.6
22	Kohora	Kathpora	26	37	22.3	93	22	20.9
23	Kohora	Kerasing	26	36	38.8	93	19	20.6
24	Kohora	Mihimukh	26	36	49.4	93	22	27.0
25	Bagori	Bahubeel	26	35	50	93	13	38.2
26	Bagori	Bimoli	26	37	45.6	93	18	9
27	Bagori	Donga	26	35	34.2	93	17	49
28	Bagori	Murphuloni	26	35	8.7	93	14	44.8
29	Bagori	Rowmari	26	35	16.7	93	17	31.3

Table S14. The number of faecal samples collected for mega and meso-herbivores during 2013-15 in Kaziranga National Park, Assam.

Species	Year	Month	Season	Sample collected	Composite samples	DOC	Forest Range			Habitat		
							Agoratoli	Kohora	Bagori	SG	TG	WL
Greater One-horned Rhino	2013	Nov	Dry	25	5	4	15	10	0	17	8	0
	2013	Dec	Dry	25	5	3	10	10	5	14	3	8
	2014	Jan	Dry	25	5	4	20	0	5	20	0	5
	2014	Feb	Dry	25	5	3	5	10	10	11	7	7
	2014	Mar	Dry	25	5	4	10	15	0	8	12	5
	2014	Apr	Wet	25	5	3	20	0	5	19	2	4
	2014	May	Wet	25	5	3	10	10	5	11	5	9
	2014	Nov	Dry	25	5	4	5	10	10	10	9	6
	2014	Dec	Dry	25	5	3	10	10	5	0	13	12
	2015	Jan	Dry	25	5	3	5	10	10	6	12	7
	2015	Feb	Dry	25	5	3	5	15	5	12	10	3
	2015	Mar	Dry	25	5	4	5	10	10	5	10	10
	2015	Apr	Wet	25	5	3	10	5	10	13	5	7
	2015	May	Wet	25	5	3	5	15	5	7	8	10
	**Overall**			**350**	**70**	**47**	**135**	**130**	**85**	**153**	**104**	**93**
Asian Elephant	2013	Nov	Dry	25	5	4	15	10	0	14	7	4
	2013	Dec	Dry	25	5	5	5	10	10	9	15	1
	2014	Jan	Dry	25	5	3	20	0	5	11	8	6
	2014	Feb	Dry	25	5	4	5	10	10	9	11	5
	2014	Mar	Dry	25	5	5	10	10	5	9	8	8
	2014	Apr	Wet	25	5	4	10	5	10	5	10	10
	2014	May	Wet	25	5	5	10	10	5	16	2	7
	2014	Nov	Dry	25	5	3	15	5	5	16	6	3
	2014	Dec	Dry	25	5	5	15	5	5	12	6	7
	2015	Jan	Dry	25	5	4	10	10	5	8	12	5
	2015	Feb	Dry	25	5	4	10	10	5	16	6	3
	2015	Mar	Dry	25	5	4	5	10	10	9	9	7
	2015	Apr	Wet	25	5	4	5	10	10	10	10	5
	2015	May	Wet	25	5	2	10	15	0	7	5	13
	**Overall**			**350**	**70**	**56**	**145**	**120**	**85**	**151**	**115**	**84**
Asiatic Wild Buffalo	2013	Nov	Dry	25	5	6	15	10	0	12	13	0
	2013	Dec	Dry	25	5	5	5	10	10	11	9	5
	2014	Jan	Dry	25	5	4	20	0	5	19	1	5
	2014	Feb	Dry	25	5	4	5	10	10	10	15	0
	2014	Mar	Dry	25	5	5	10	10	5	15	5	5
	2014	Apr	Wet	25	5	4	10	10	5	10	15	0
	2014	May	Wet	15	3	3	10	5	0	15	0	0
	2014	Nov	Dry	25	5	3	20	0	5	19	6	0
	2014	Dec	Dry	25	5	4	15	5	5	17	5	3
	2015	Jan	Dry	25	5	4	10	10	5	17	6	2
	2015	Feb	Dry	25	5	4	10	5	10	20	5	0
	2015	Mar	Dry	25	5	4	5	10	10	18	7	0
	2015	Apr	Wet	25	5	3	0	15	10	8	10	7
	2015	May	Wet	10	2	2	5	5	0	8	0	2
	**Overall**			**325**	**65**	**55**	**140**	**105**	**80**	**199**	**97**	**29**
Swamp Deer	2013	Nov	Dry	25	5	3	15	10	0	17	8	0
	2013	Dec	Dry	25	5	4	15	5	5	16	4	5
	2014	Jan	Dry	25	5	4	20	0	5	23	2	0
	2014	Feb	Dry	25	5	4	10	5	10	20	5	0
	2014	Mar	Dry	25	5	3	10	10	5	21	4	0
	2014	Apr	Wet	25	5	4	15	5	5	20	5	0
	2014	May	Wet	15	3	3	10	5	0	15	0	0

Species	Year	Month	Season	Sample collected	Composite samples	DOC	Forest Range			Habitat		
							Agoratoli	Kohora	Bagori	SG	TG	WL
	2014	Nov	Dry	25	5	4	5	10	10	20	5	0
	2014	Dec	Dry	25	5	3	10	10	5	24	1	0
	2015	Jan	Dry	25	5	3	10	5	10	24	1	0
	2015	Feb	Dry	25	5	5	15	5	5	22	3	0
	2015	Mar	Dry	25	5	4	5	10	10	15	10	0
	2015	Apr	Wet	25	5	4	10	10	5	25	0	0
	2015	May	Wet	10	2	2	5	5	0	5	5	0
	**Overall**			**325**	**65**	**50**	**155**	**95**	**75**	**267**	**53**	**5**
Hog Deer	2013	Nov	Dry	25	5	5	15	10	0	17	8	0
	2013	Dec	Dry	25	5	4	10	10	5	16	5	4
	2014	Jan	Dry	25	5	4	15	0	10	25	0	0
	2014	Feb	Dry	25	5	3	5	10	10	19	6	0
	2014	Mar	Dry	25	5	2	10	10	5	16	9	0
	2014	Apr	Wet	25	5	5	10	5	10	21	4	0
	2014	May	Wet	15	3	3	10	5	0	15	0	0
	2014	Nov	Dry	25	5	4	15	10	0	15	10	0
	2014	Dec	Dry	25	5	4	5	15	5	20	5	0
	2015	Jan	Dry	25	5	3	10	5	10	24	1	0
	2015	Feb	Dry	25	5	4	15	5	5	20	5	0
	2015	Mar	Dry	25	5	4	10	5	10	21	4	0
	2015	Apr	Wet	25	5	4	5	10	10	21	4	0
	2015	May	Wet	10	2	2	5	5	0	6	4	0
	**Overall**			**325**	**65**	**51**	**140**	**105**	**80**	**256**	**65**	**4**
Sambar	2013	Nov	Dry	25	5	5	15	10	0	5	6	14
	2013	Dec	Dry	25	5	4	10	5	10	6	2	17
	2014	Jan	Dry	25	5	4	15	0	10	6	5	14
	2014	Feb	Dry	25	5	4	10	10	5	9	6	10
	2014	Mar	Dry	25	5	5	5	20	0	18	6	1
	2014	Apr	Wet	25	5	3	5	15	5	8	5	12
	2014	Nov	Dry	25	5	5	10	10	5	9	5	11
	2014	Dec	Dry	25	5	5	10	10	5	10	4	11
	2015	Jan	Dry	25	5	3	5	15	5	11	12	2
	2015	Feb	Dry	25	5	4	5	15	5	5	15	5
	2015	Mar	Dry	25	5	3	10	10	5	1	11	13
	2015	Apr	Wet	25	5	3	10	15	0	10	7	8
	**Overall**			**300**	**60**	**48**	**110**	**135**	**55**	**98**	**84**	**118**

DOC- Days of collection, Nov-November, Dec, December, Jan-January, Feb-February, Mar- March, Apr-April,
SG- short grassland, TG- tall grassland, WL- woodland

Table S15. The methods used for estimation of the nutrient content of major forage utilized by mega and meso-herbivores during 2013-15 in Kaziranga National Park, Assam.

**Nutrient Parameters**	**Method Used**	**Instrument used**	**Reference**
Ash content	Combustion (550 °C) method	Furnace	1, 2
Crude Protein	Kjeldahl method	Kjeldahl Nitrogen Analyzer (Foss Analytical 2003a)	1, 2
Acid Detergent Fibre	Van Soest detergent method	Fiber Analyzer (Foss Analytical 2003b and 2003c)	2, 3
Acid Detergent Lignin	Van Soest detergent method	Fiber Analyzer (Foss Analytical 2003b and 2003c)	2, 3
Neutral Detergent Fibre	Van Soest detergent method	Fiber Analyzer (Foss Analytical 2003b and 2003c)	2, 3
Calcium and Magnesium	Atomic Absorption Spectrophotometry	Atomic Absorption Spectrophotometer	1, 2
Sodium and Potassium	Flame photometry	Systronics flame photometer (128)	1, 2
Phosphorous	Vanadomolybdo phosphoric Acid Colorimetric method	UV Visible Spectrophotometer (Varian's Carry-100)	1, 2

1-AOAC, 1990; 2- Chaturvedi & Sankar, 2006; 3- Goering & Van Soest, 1970

Crude Protein- The Auto Kjeldahl nitrogen analyser consists of two separate units, the block digester and the distillation titration assembly. For digestion, 0.5 gm of oven dried sample was mixed with a catalyst ($CuSO_4$ + K_2SO_4) and 10 ml of concentrated sulphuric acid. Then this mixture was digested in digestion chamber at 420 °C for five to six hours in the block digester. The digested samples were transferred into the distillation titration assembly where the ammonia generated during distillation was absorbed in 2% Boric acid, which was then titrated against 0.01N sulphuric acid. A blank (without sample) was run for each set of samples. The percent crude protein was calculated following Chaturvedi & Sankar (2006).

Minerals- For analysis of minerals, the Di-acid digestion technique was used in which 0.5 gm of dried and processed plant samples were weighed and mixed with nitric acid and perchloric acid (9ml: 4ml). The mixture was then digested at 180 °C to 200 °C for about 5-6 hours in digestion chamber. After cooling down, the mixture was filtered in a volumetric flask with double distilled water up to the volume of 100 ml (Chaturvedi & Sankar, 2006). In Atomic Absorption Spectrophotometer (AAS), the Di-acid digested mixture was used to estimate its Ca and Mg content. Similarly, in Systronics flame photometer (128) the Di-acid digested mixture was used to estimate its Na and K content. Both AAS and flame photometer were calibrated using standard solutions of the elements to be examined. In UV Visible Spectrophotometer (Varian's Carry-100), Di-acid digested mixture was used to estimate its P content. For data analysis, the parts per million (ppm) readings recorded for mineral parameters were converted into percentages (Allen et al., 1974).

References

1. Association of Official Agricultural Chemists (AOAC). *Official methods of analysis of the Association of Official Analytical Chemists*. (Association of official analytical chemists, Inc., 1990).
2. Chaturvedi R. K. & Sankar K. *Laboratory manual for the physico-chemical analysis of soil, water and plant*. Wildlife Institute of India, Dehradun, 1-97 (2006).
3. Goering, H. K., & Van Soest, P. J. *Forage fiber analyses (Apparatus, reagents, procedures, and some applications)*: Agricultural Handbook No. 379 (1970).
4. Allen, S. E., Grimshaw, H. M., Parkinson, J. A., and Quarmby, C. *Chemical analysis of ecological materials*. (Second eds. Allen, S. E.), 1-368 (Oxford, UK, Blackwell Scientific Publications ,1974).
